# Tumor Cell-Surface Binding of Immune Stimulating Polymeric Glyco-Adjuvant via Cysteine-Reactive Pyridyl Disulfide Promotes Antitumor Immunity

**DOI:** 10.1021/acscentsci.2c00704

**Published:** 2022-10-07

**Authors:** Anna J. Slezak, Aslan Mansurov, Michal M. Raczy, Kevin Chang, Aaron T. Alpar, Abigail L. Lauterbach, Rachel P. Wallace, Rachel K. Weathered, Jorge E.G. Medellin, Claudia Battistella, Laura T. Gray, Tiffany M. Marchell, Suzana Gomes, Melody A. Swartz, Jeffrey A. Hubbell

**Affiliations:** † Pritzker School of Molecular Engineering, University of Chicago, Chicago, Illinois 60637, United States; ‡ Committee on Immunology, University of Chicago, Chicago, Illinois 60637, United States; § Ben May Department for Cancer Research, University of Chicago, Chicago, Illinois 60637, United States; ∥ Committee on Cancer Biology, University of Chicago, Chicago, Illinois 60637, United States

## Abstract

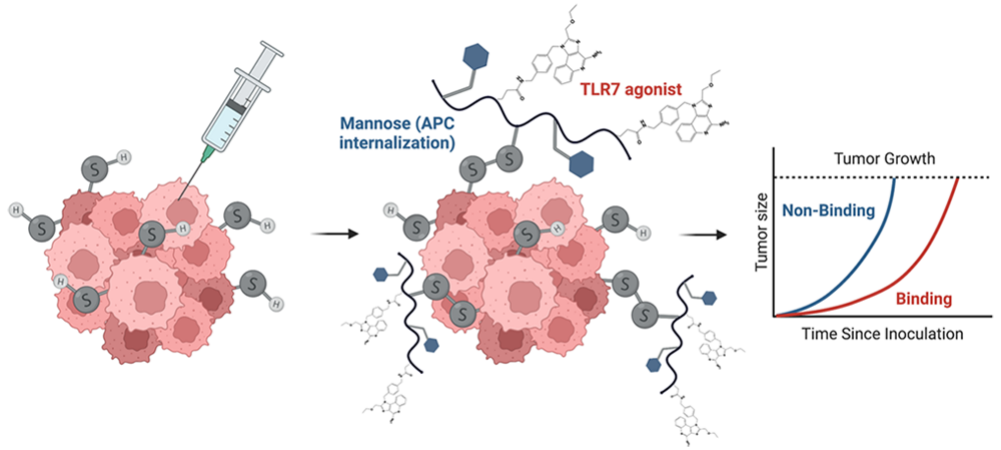

Immune stimulating agents like Toll-like receptor 7 (TLR7) agonists induce potent antitumor immunity but are limited in their therapeutic window due to off-target immune activation. Here, we developed a polymeric delivery platform that binds excess unpaired cysteines on tumor cell surfaces and debris to adjuvant tumor neoantigens as an *in situ* vaccine. The metabolic and enzymatic dysregulation in the tumor microenvironment produces these exofacial free thiols, which can undergo efficient disulfide exchange with thiol-reactive pyridyl disulfide moieties upon intratumoral injection. These functional monomers are incorporated into a copolymer with pendant mannose groups and TLR7 agonists to target both antigen and adjuvant to antigen presenting cells. When tethered in the tumor, the polymeric glyco-adjuvant induces a robust antitumor response and prolongs survival of tumor-bearing mice, including in checkpoint-resistant B16F10 melanoma. The construct additionally reduces systemic toxicity associated with clinically relevant small molecule TLR7 agonists.

## Abstract 2

Thiol-reactive polymeric adjuvant covalently binds unpaired cysteines upon intratumoral injection to adjuvant tumor cells and debris for immune recognition and subsequent slowed tumor growth.

## Introduction

While the features of solid tumors vary considerably, some physiological properties are common and create a characteristic microenvironment for tumor growth and proliferation.^1^ Rapid, disordered metabolism leads to a harsh environment with unique chemical characteristics including hypoxia and acidosis.^2,3^ This also leads to physical disorganization in the form of disordered and hyperpermeable vasculature and interstitial hypertension.^4^ For rapid growth and immune evasion to occur, cancer cells make adaptive changes to protein expression, especially to those involved in metabolism and extracellular matrix degradation, including p53, hypoxia-inducible factor-1α, and matrix metalloproteases.^5,6^ Together, these create a characteristic tumor microenvironment.

While the advent of immune checkpoint inhibitors has transformed the field of cancer immunotherapy, their suboptimal response rate in certain tumors, including melanoma and breast cancer, leaves room for the development of complementary therapeutics.^7−9^ Immune stimulating agents such as Toll-like receptor (TLR) agonists can activate the innate immune system and potentiate antitumor immunity.^10,11^ In particular, we are interested in the delivery of agonists to TLR7 and TLR8, whose natural ligand is single-stranded RNA.^12^ A small molecule mimetic, imiquimod, was first clinically approved for topical administration in 1997.^13^ Since then, new iterations of TLR7 and TLR8 agonists have been developed, including resquimod (R848), motolimod (VTX-2337), and others, which offer improved solubility and more pronounced downstream affects.^14,15^ However, their clinical translation has been limited due to a narrow therapeutic window.^16,17^ Improving the tolerability and efficacy of such agonists using chemical modifications to localize them in the tumor microenvironment can broaden their clinical applicability.

Given the dysregulated metabolism of the tumor microenvironment, we hypothesized that the metabolic stress of tumor growth produces an excess of unpaired cysteines on cell-surface proteins relative to other cells of the body. It has been documented that the redox properties of the tumor microenvironment itself vary based on aggressiveness and stage of the tumor and can alter its responsiveness to some drugs.^18−21^ We envisioned exploiting the resulting excess of free thiols in the tumor microenvironment for a drug delivery application. Consequently, we created a delivery platform using a thiol-specific reactive moiety, pyridyl disulfide, for conjugation to the tumor cell surface to achieve prolonged tumor retention. We chose to deliver a polymeric glyco-adjuvant previously developed in our laboratory, p(Man-TLR7), which showed strong efficacy as a vaccinal adjuvant.^22^ We now pivoted to an immuno-oncology application to adjuvant the tumor cell surface and the neoantigens contained therein and demonstrated that disulfide binding upon intratumoral injection created an *in situ* cancer vaccine, inducing strong therapeutic antitumor immunity as a monotherapy and in combination with checkpoint inhibition.

## Results

### Multivalent, Cysteine-Reactive Polymers Can Be Synthesized via PET-RAFT

**Figure 1:**
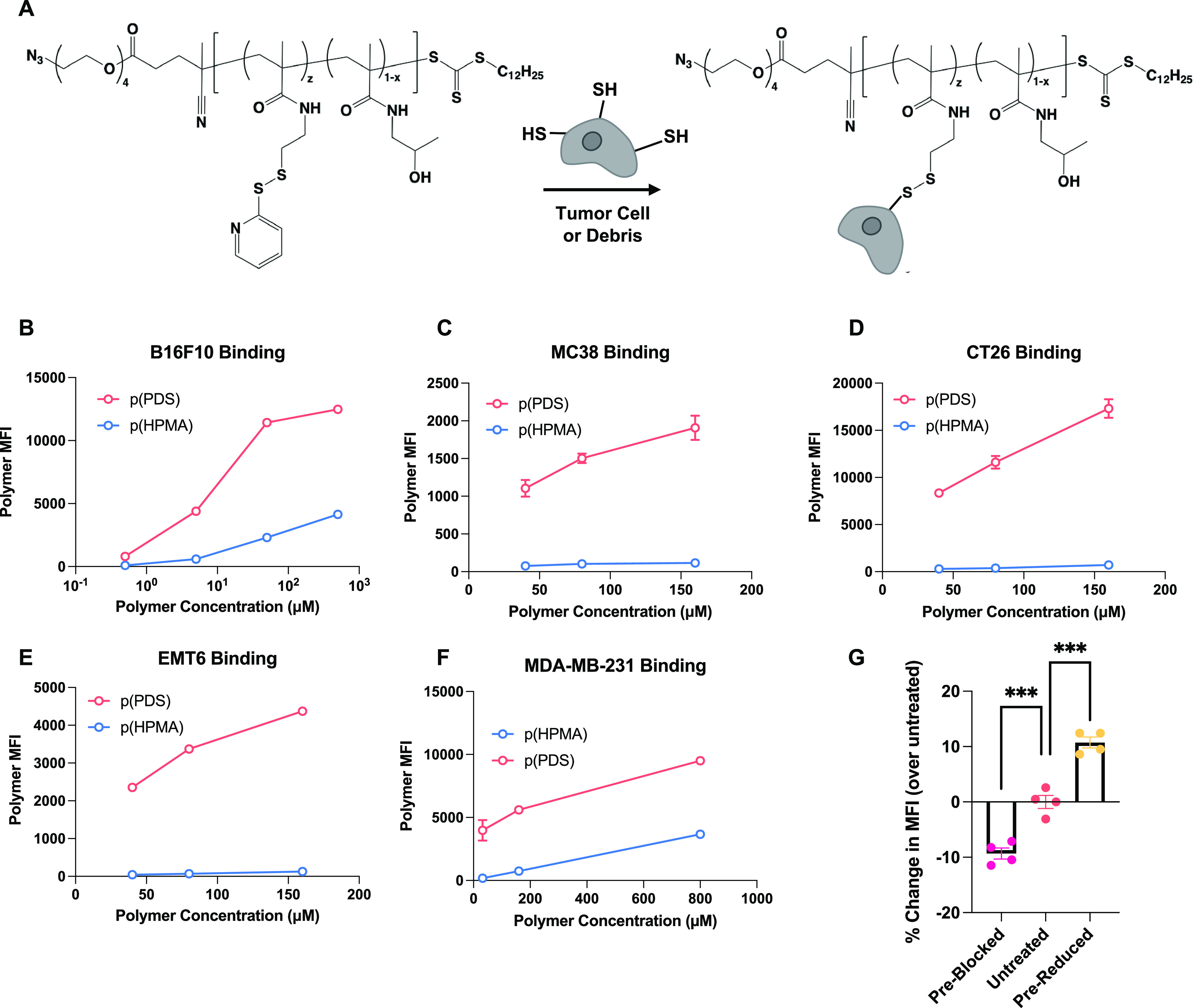
Polymeric PDS binds tumor cells *in vitro.* (A) Schematic of PDS disulfide exchange with exofacial protein thiols on tumor cells, enabling *in situ* adjuvant conjugation. (B) Mean fluorescence intensity (MFI) data of concentration-dependent binding of fluorescently labeled p(PDS) or “spacer” only p(HPMA) to B16F10 cells as quantified by flow cytometry. The same assay was repeated on (C) EMT6, (D) CT26, (E) MC38, and (F) MDA-MB-231 cells (mean ± SEM; *n* = 3). (G) Fold change in binding (relative to untreated cells) of fluorescently labeled p(PDS) to B16F10 cells after preblocking exofacial protein thiols with N-ethyl maleimide or prereducing extracellular disulfide bonds with TCEP (mean ± SEM; *n* = 4). Statistical analysis was done using ordinary one-way analysis of variance. ***p < 0.001.

Pyridyl disulfide (PDS) moieties are useful in the drug delivery field due to their rapid and efficient thiol–disulfide exchange reaction, which can be used to reversibly conjugate therapeutics or fabricate materials for *in situ* binding.^23−25^ We developed a synthesis scheme for a PDS-containing methacrylamide monomer.^26^ In order to attain high conversion polymerization without significant cross-linking, we employed photoinduced electron/energy transfer-reversible addition–fragmentation chain transfer (PET-RAFT). This method uses a photoredox catalyst, eosin Y in our case, to initiate controlled free radical polymerization with high conversion and low dispersity.^27,28^ We copolymerized our PDS monomer with the bioinert, water-soluble, “spacer” monomer 2-hydroxypropyl methacrylamide (HPMA). This strategy predictably yielded statistical copolymers (referred to as p(PDS)) of desired molecular weight and composition (Figure S1). These functional polymers reacted efficiently with unpaired cysteines on tumor cell-surface proteins (Figure 1a). In order to confirm the functional activity of p(PDS), we reduced the polymer with β-mercaptoethanol and monitored the production of the leaving group, 2-mercaptopyridine, which has a characteristic absorbance at 343 nm^29^ (Figure S2).

### Pyridyl Disulfide Mediates Cell-Surface Binding and Tumor Retention

Once we confirmed the production of uniform, reactive, PDS-containing polymers, we validated their binding ability to tumor cells *in vitro*. After incubation at 4 °C to inhibit endocytosis, we quantified, via flow cytometry, that p(PDS) bound B16F10 melanoma cells significantly better than a molecular weight-matched p(HPMA) (nonbinding control) (Figure 1b). This result was validated using other murine cancer cell lines, including MC38 and CT26 colon carcinoma and EMT6 mammary carcinoma (Figure 1c–e) and human breast cancer cell line MDA-MB-231 (Figure 1f). In order to ensure that covalent binding to exofacial protein thiols mediated the increase in binding, we first pretreated B16F10 cells with N-ethyl maleimide to block free thiols or tris(2-carboxyethyl)phosphine (TCEP) to reduce disulfide bonds into free thiols before the polymer incubation. As expected, preblocking thiols prevented polymer binding and reduced mean fluorescence intensity (MFI) (Figure 1g). Likewise, prereducing thiols increased reactive sites for polymer binding and thus increased MFI relative to the untreated samples.

**Figure 2:**
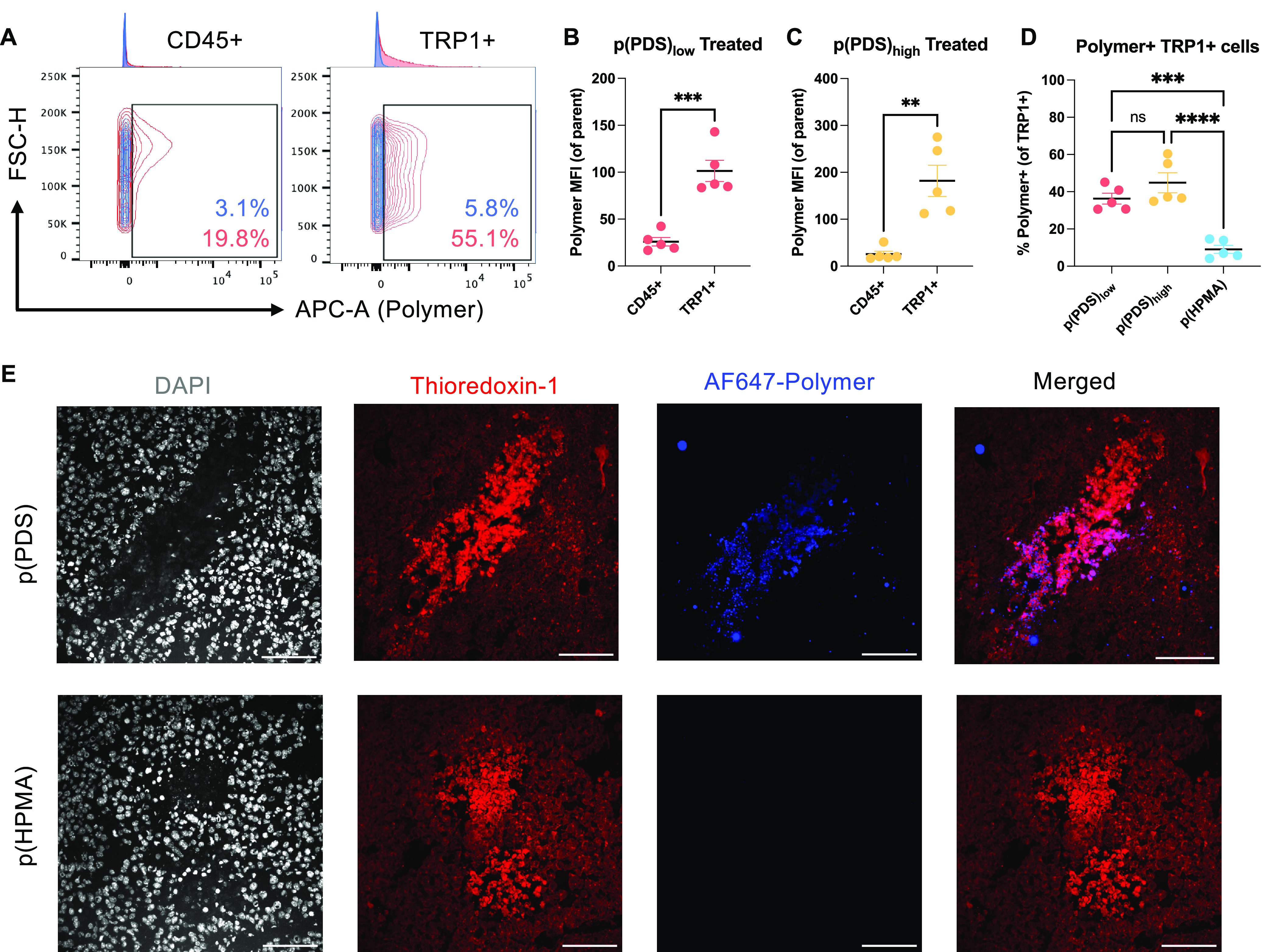
PDS polymers preferentially bind tumor cells *in vivo*. Mice bearing established B16F10 tumors were injected intratumorally with fluorescently labeled p(PDS) or p(HPMA). After 3 h, tumors were digested and stained for flow cytometry. (A) Representative contour flow cytometry plots, comparing the frequency of polymer^+^ cells of CD45^+^ immune cells or TRP1^+^ tumor cells between mice treated with p(PDS) in red or p(HPMA) in blue. Both polymers with (B) low or (C) high PDS content preferentially bound TRP1^+^ tumor cells over CD45^+^ immune cells, as quantified by the polymer median fluorescence intensity (MFI) of the parent population. (D) Both PDS-containing polymers bound a higher frequency of TRP1^+^ tumor cells than the nonbinding p(HPMA) control (mean ± SEM; *n* = 5). Experiment was repeated twice with similar results. Representative data shown. (E) Alternatively, after 6 h, tumors were harvested and analyzed by fluorescence microscopy. Tissues were stained with DAPI (gray) and AF594 conjugated antithioredoxin-1 (red). Merged panel contains only thioredoxin-1 and polymer (blue) channels. Scale bars, 100 μm. Representative images of *n* = 2 biological replicates with *n* = 3 technical replicates. Statistical analyses were performed using unpaired *t*-tests (B,C) and ordinary one-way analysis of variance (D). **p < 0.01; ***p < 0.001; ****p < 0.0001; ns = not significant.

Binding was next evaluated *in vivo* by two methods. First, we quantified binding to different cell populations after intratumoral injection into B16F10 melanoma-bearing mice. We observed that TRP1^+^ B16F10 cells on average bound more polymer than CD45^+^ immune cells. This result was consistent between mice treated with polymer with low or high PDS content (Figures 2a–c and S3). Additionally, both PDS polymers were more likely to bind TRP1^+^ tumor cells than was the nonbinding, HPMA-only control, as evidenced by a higher frequency of polymer^+^ cells (Figure 2d). We also confirmed intratumoral retention via fluorescence microscopy (Figure 2e). Six hours after injection of fluorescently labeled polymer, we observed retention of p(PDS) but not the nonbinding control p(HPMA). Interestingly, the polymer colocalized with thioredoxin-1, which is a small protein with a redox-active disulfide/dithiol that plays a vital role in redox signaling by reducing oxidized cysteine residues and cleaving disulfide bonds.^30^ Thioredoxin has been reported to be upregulated in many aggressive human cancer types, and its expression is correlated with worse patient outcomes.^31,32^ Thioredoxin-1 staining was particularly strong in regions with low viability (as assessed by DAPI staining), suggesting that intracellular thioredoxin-1, which is detectable inside B16F10 cells (Figure S4), may be released through cell death. This result suggests thioredoxin-1 upregulation facilitates intratumoral polymer binding.

### PDS Incorporation Facilitates Tumor Cell Binding and Maintains *In Vitro* Activity of p(Man-TLR7)

**Figure 3:**
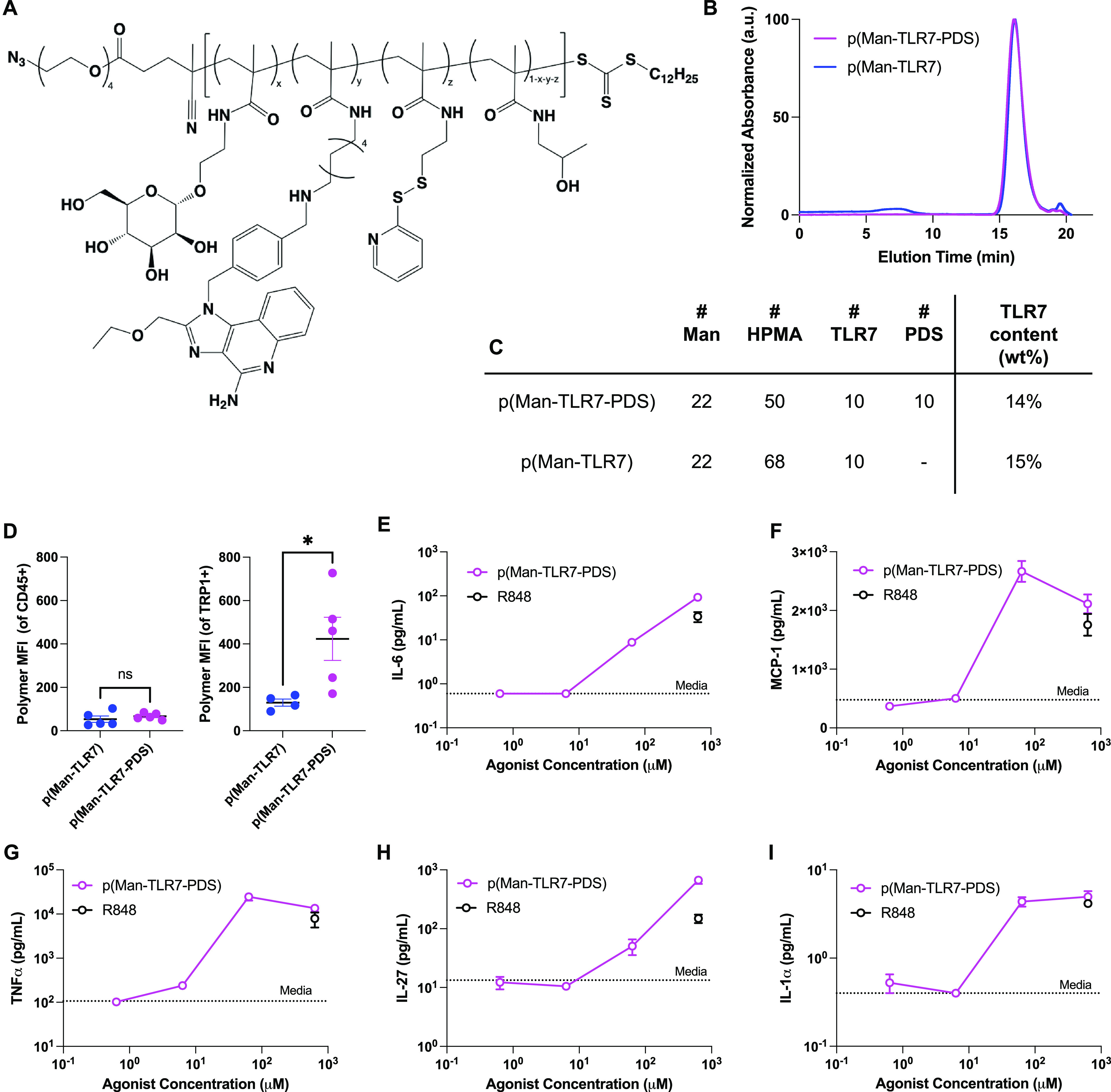
Synthesis and characterization of p(Man-TLR7-PDS). (A) Structure of full p(Man-TLR7-PDS) statistical copolymer. (B) GPC elution profile of p(Man-TLR7-PDS) and nonbinding control p(Man-TLR7). (C) Table of monomer feed ratios relative to chain transfer agent used to synthesize p(Man-TLR7-PDS) and p(Man-TLR7) and weight percent TLR7 monomer of resulting polymers. (D) Mice bearing established B16F10 tumors were injected intratumorally with fluorescently conjugated p(Man-TLR7-PDS) or p(Man-TLR7). After 3 h, tumors were digested and stained for flow cytometry. Full p(Man-TLR7-PDS) preferentially binds TRP1^+^ tumor cells over CD45^+^ immune cells, as quantified by polymer median fluorescence intensity (MFI). Nonbinding p(Man-TLR7) showed significantly less binding to tumor cells than p(Man-TLR7-PDS). (E–I) Concentration-dependent secretion of proinflammatory cytokines by RAW 264.7 cells in response to stimulation with p(Man-TLR7-PDS) including (E) IL-6, (F) MCP-1, (G) TNFα, (H) IL-27, or (I) IL-1α with control TLR7/8 agonist R848 (mean ± SEM; *n* = 3). Statistical analyses were performed using unpaired *t*-tests. **p < 0.01; ***p < 0.001; ns = not significant.

Our lab previously reported a polymeric glyco-adjuvant, p(Man-TLR7), which elicited robust humoral and cellular immunity in a vaccine context;^22,33^ the polymer is composed of a mannosylated methacrylamide monomer intended to bind a mannose receptor on antigen-presenting cells (APCs) and trigger endocytosis and an imidazoquinoline TLR7 agonist methacrylamide monomer for APC activation once inside the endosome. Now, inspired by the success of other TLR7 agonists in cancer immunotherapy,^34,35^ we evaluated the antitumor efficacy of the glyco-adjuvant. To evaluate the applicability of our delivery platform, we incorporated PDS monomers into p(Man-TLR7) to produce a thiol-binding polymeric glyco-adjuvant, p(Man-TLR7-PDS) (Figure 3a). For this polymer, we selected feed ratios of the TLR7 and mannose monomers such that the weight percent of the TLR7 agonist would be 15%, which was previously confirmed to be optimally active while maintaining water solublility.^22^ Low dispersity, uniform polymers with similar size were synthesized via PET-RAFT (Figure 3b). The PDS monomer ratio was comparable to the p(PDS)_high_ polymer from binding studies (Figure 3c).

Because our functional polymer, p(Man-TLR7-PDS), could also hypothetically bind mannose receptor or other c-type lectins on tumor cell surfaces,^36,37^ we verified that mannose monomer incorporation into the polymer did not affect PDS-mediated tumor cell binding, with or without mannose preincubation (Figures S5 and S6). We found that the full immune-functional copolymer bound tumor cell surfaces in an exclusively thiol-dependent manner. Further, we replicated the *in vivo* cell binding flow cytometry experiment (from Figures 2a–c and S3) to determine whether mannose and TLR7 agonist incorporation affected PDS-mediated cell binding and uptake. Our results were consistent with the previous study and demonstrated low polymer binding to CD45^+^ immune cells and significantly higher binding of only PDS-containing p(Man-TLR7-PDS), but not p(Man-TLR7), to TRP1^+^ B16F10 tumor cells (Figure 3d).

Our previous work with p(Man-TLR7) focused on *in vitro* activity on murine bone marrow derived dendritic cells (BMDCs) (Figure S7). Here, to demonstrate immune agonization by the copolymer, we used macrophages as a model, also noting the high frequency tumor-associated macrophages expressing macrophage mannose receptor CD206.^38,39^ We stimulated macrophage-like RAW 264.7 cells and observed a dose-dependent increase in proinflammatory cytokines that are downstream of TLR7 signaling (Figure 3e–i).^40^ As a positive control for our assay, we included a well-known TLR7/8 agonist R848 at our top concentration. We also confirmed that polymer uptake by antigen presenting cells is mediated by mannose, not PDS, using a BMDC uptake experiment (Figure S8). This can be explained by the high affinity of mannose for CD206 and other mannose receptors or by the relative lack of unpaired cysteines on nontumor cell surfaces. We also verified that the polymer did not have inherent cytotoxicity to BMDCs or B16F10 melanoma cells (Figures S9 and S10).

### p(Man-TLR7-PDS) Significantly Slows Tumor Growth in Orthotopic Murine Cancers

**Figure 4:**
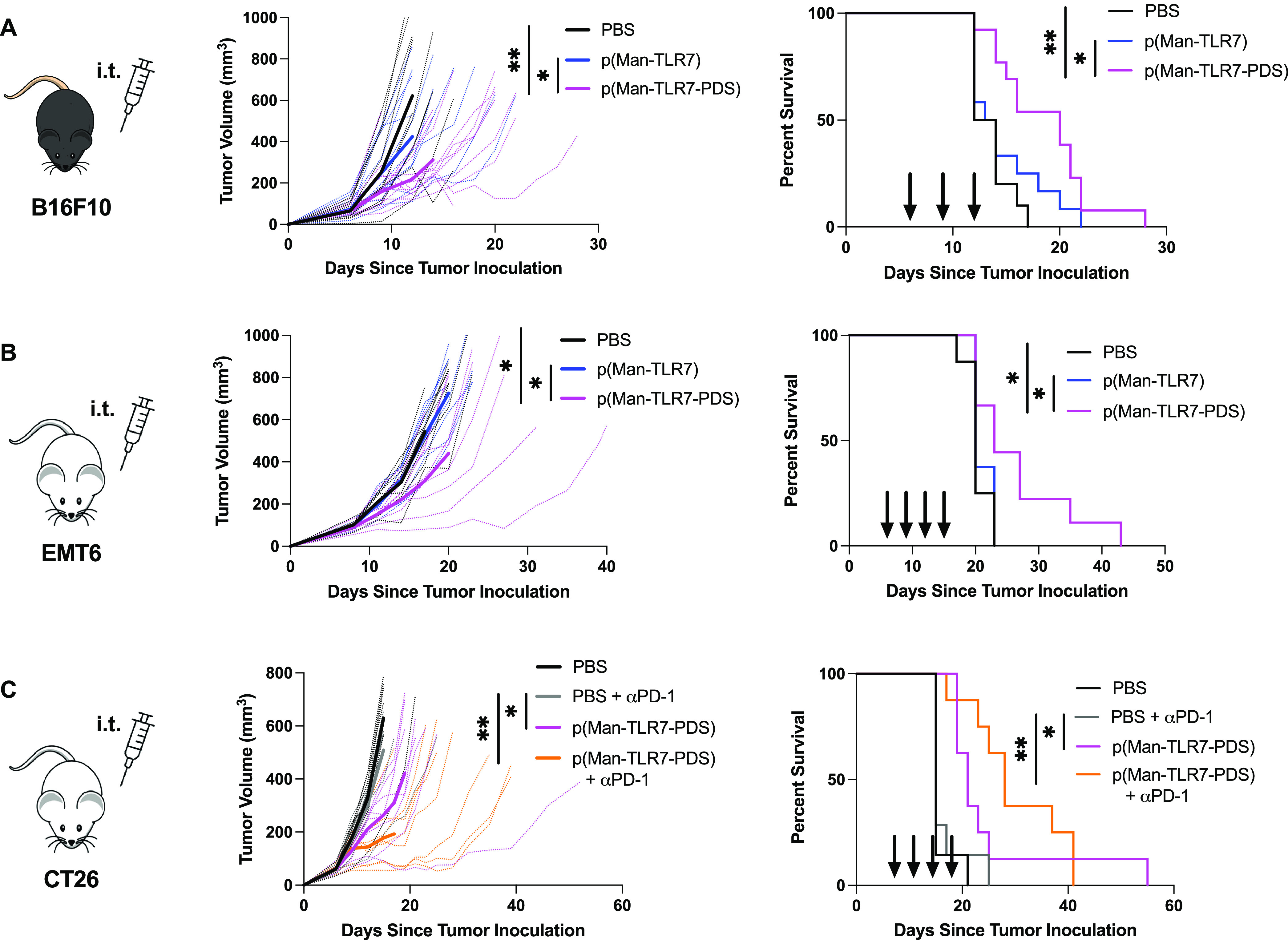
p(Man-TLR7-PDS) significantly slows tumor growth and prolongs survival in various murine cancer models. (A) Mice were inoculated intradermally on the left shoulder with 500 000 B16F10 melanoma cells and treated intratumorally with 40 μg of TLR7 monomer equivalent p(Man-TLR7-PDS) (*n* = 13), p(Man-TLR7) (*n* = 12), or vehicle control (PBS, *n* = 10) on days 6, 9, and 12 after tumor inoculation. Data are compiled from two independent experiments. (B) Mice were inoculated with 500 000 EMT6 mammary carcinoma cells into the left mammary fat pad and were treated intratumorally with 40 μg of TLR7 monomer equivalent p(Man-TLR7-PDS) (*n* = 9), nonbinding p(Man-TLR7) (*n* = 8), or vehicle control (PBS, *n* = 9) on days 6, 9, 12, and 15 after tumor inoculation. (C) Mice were inoculated subcutaneously on the left shoulder with 500 000 CT26 colon carcinoma cells and treated with PBS (*n* = 7), 100 μg of anti-PD-1 (*n* = 7), 40 μg of TLR7 monomer equivalent polymer (*n* = 8), or polymer + anti-PD-1 (*n* = 8) on days 6, 9, 12, and 15 after tumor inoculation. Polymer or PBS were administered intratumorally, and anti-PD-1 was administered intraperitoneally. For all experiments, shown are individual (thin dotted lines) and mean (thick solid lines) tumor growth curves and survival plots. Arrows denote treatment days on survival plots. Statistical analyses were performed using unpaired *t*-tests of tumor measurements on the last day all mice are surviving and log-rank (Mantel-Cox) curve comparison for survival.

To evaluate the superiority of the thiol-binding p(Man-TLR7-PDS) over the nonbinding p(Man-TLR7), we injected B16F10 melanoma-bearing mice intratumorally with the polymers, with the dose based on TLR7 content. We observed slowing of tumor growth and prolongation of survival in mice treated with the binding polymer but not the nonbinding control polymer (Figure 4a). These results were also validated in orthotopic EMT6, an immune-excluded triple-negative breast cancer model (Figure 4b), thus demonstrating the broad applicability of the tumor-agnostic delivery platform. This supports our hypothesis that PDS-mediated disulfide anchoring increases the antitumor efficacy of cancer immunotherapies. As a control, we also studied the antitumor efficacy of nonadjuvanted p(Man-PDS) (Figure S11). The polymer provided no tumor protection, verifying that the antitumor efficacy of the polymer is TLR7 agonist-dependent. Additionally, we compared the antitumor efficacy of two fully separate batches of p(Man-TLR7-PDS) to ensure low batch-to-batch variability (Figure S12).

### p(Man-TLR7-PDS) Improves Efficacy of Checkpoint Inhibitors

To demonstrate that our technology can enhance the efficacy of immune checkpoint blockade therapy, we evaluated its efficacy in combination with the immune checkpoint inhibitor anti-PD-1, which is the most commonly used form of immunotherapy.^41^ We selected CT26 colon carcinoma as our disease model due to its documented low-to-moderate response rate to checkpoint inhibitors.^42^ In this model, we dosed p(Man-TLR7-PDS) intratumorally and anti-PD-1 systemically. As expected, mice treated with anti-PD-1 alone had comparable outcomes to the vehicle-only control group (Figure 4c). Therapy with p(Man-TLR7-PDS) alone demonstrated significantly higher efficacy over anti-PD-1 monotherapy, but the effect of the combination therapy was even more pronounced. This result is promising for the translational potential of the technology, as it can synergize with checkpoint inhibitors to treat anti-PD-1-resistant tumors.

### p(Man-TLR7-PDS) Eradicates Established MC38 Colon Carcinoma

**Figure 5:**
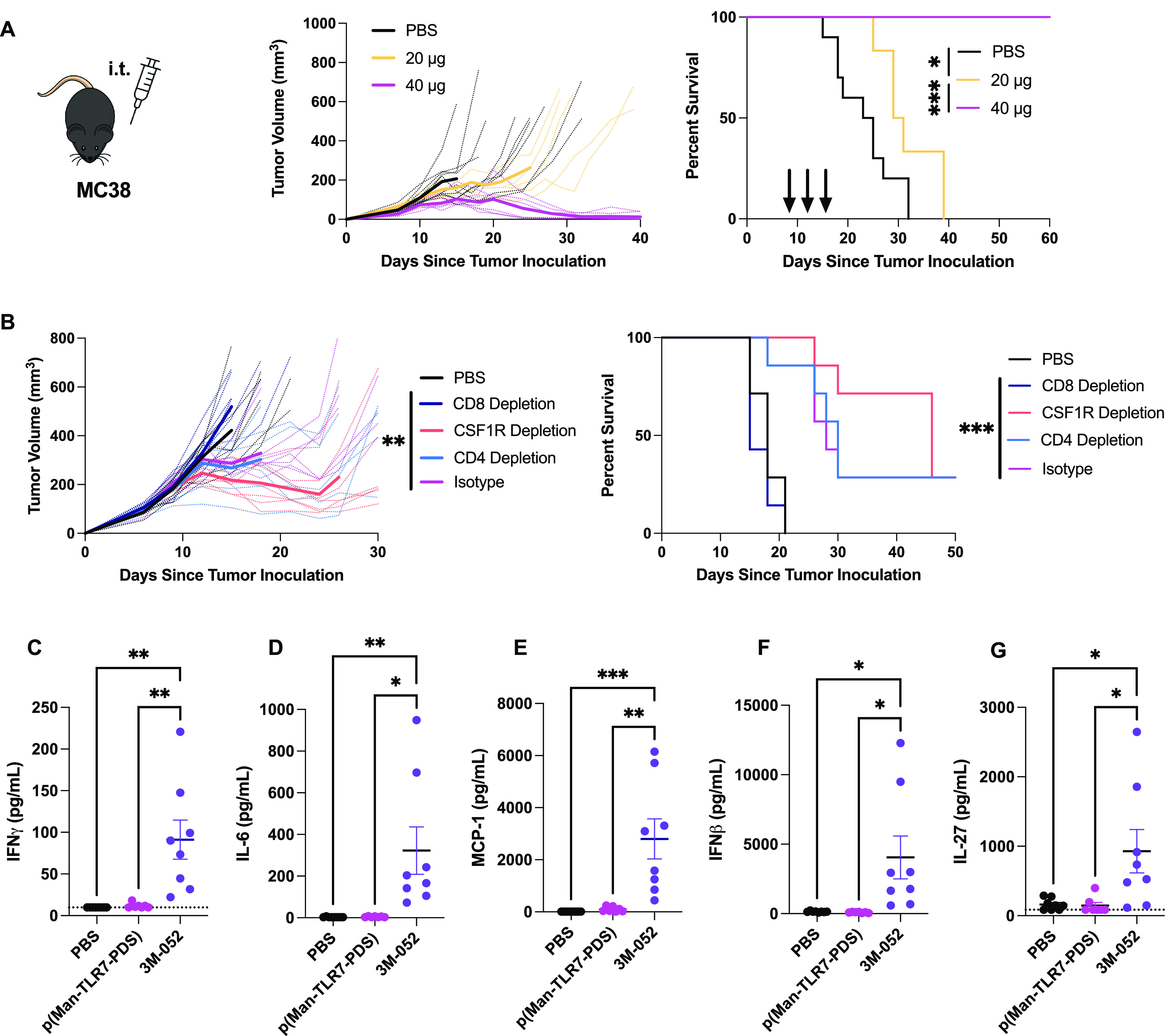
p(Man-TLR7-PDS) effectively eradicates MC38 colon carcinoma and limits toxicity of TLR7/8 agonist therapeutics. (A) Mice were inoculated subcutaneously with 500 000 MC38 colon carcinoma cells into the left shoulder and injected with 20 or 40 μg of TLR7 monomer equivalent polymer on days 7, 10, and 13 after tumor inoculation. Shown are individual (thin dotted lines) and mean (thick solid lines) tumor growth curves and survival plots. (B) Mice were inoculated subcutaneously with 500 000 MC38 colon carcinoma cells into the left shoulder. CD4, CD8, CSF1R, or isotype control depletion antibodies were administered as described in methods. Mice were treated intratumorally with 40 μg of TLR7 monomer equivalent polymer or vehicle control (PBS) every 3 days for a total of three injections once tumors reached a volume of 100 mm^3^ (*n* = 7 for all groups). Shown are individual (thin dotted lines) and mean (thick solid lines) tumor growth curves and survival plots. (C–G) MC38 tumor-bearing mice were injected intratumorally with p(Man-TLR7-PDS), control TLR7/8 agonist 3M-052, or PBS. Six hours after injection, sera were collected and analyzed for proinflammatory cytokines, including (C) IFNγ, (D) IL-6, (E) MCP-1, (F) IFNβ, and (G) IL-27. Results were validated using a similar experimental design in the B16F10 melanoma model with similar results. Representative data shown. Statistical analyses were performed using unpaired *t*-tests of tumor measurements on the last day all mice are surviving (A), one-way analysis of variance on the last day all mice are surviving (B), log-rank (Mantel-Cox) curve comparison for survival (A,B), and ordinary one-way analysis of variance (C–G). *p < 0.05 **p < 0.01; ***p < 0.001, ****p < 0.0001.

We next moved to evaluate the translational potential of p(Man-TLR7-PDS). To do so, we aimed to establish dose-dependent efficacy of the technology in MC38 colon carcinoma. In this model, p(Man-TLR7-PDS) demonstrated dose-dependent antitumor control and, at the high dose, achieved a 100% survival rate (Figure 5a). We also confirmed the dose-dependency of p(Man-TLR7-PDS) in B16F10 melanoma (Figure S13).

### Antitumor Efficacy of p(Man-TLR7-PDS) Is Dependent on CD8^+^ T Cells

To evaluate which immune cell populations were mediating antitumor efficacy, we administered depletion antibodies specific for CD4, CD8α, or colony stimulating factor-1 receptor (CSF1R) to MC38 colon carcinoma-bearing mice prior to starting polymer therapy. In order to observe more significant differences, we waited until tumors reached an average size of ∼100 mm^3^. The results established a clear dependence on CD8^+^ T cells, as tumor growth for those mice was comparable to that of the PBS-treated group (Figure 5b), as is observed with many immunotherapies.^43^ Although our previous work demonstrated the importance of CD4^+^-T cell-mediated humoral response in a vaccination setting,^22^ in the tumor setting, CD4 depletion did not affect the therapeutic efficacy, likely due to depletion of regulatory T cells. Given that p(Man-TLR7-PDS) can bind both macrophages and dendritic cells (DCs), the dispensable role of macrophages, as indicated by a lack of an ameliorated response following CSF1R depletion, points to DCs in the tumor microenvironment as being the likely target for adjuvanted tumor cell material.^44^ It is known that TLR7 agonist-induced antitumor activity can be mediated through DCs.^16^ However, it is important to note that CSF1R depletion has a complex effect, as it reduces both proinflammatory M1-like macrophages as well as anti-inflammatory M2-like tumor-associated macrophages.^45^ Although p(Man-TLR7-PDS) may preferentially bind M2-like macrophages due to their high levels of CD206 expression,^46^ the interpretation of this result is complex. Nonetheless, these results established that the antitumor efficacy of p(Man-TLR7-PDS) is indispensably mediated by CD8^+^ T cells.

### Cysteine Binding Limits Systemic Inflammation Associated with TLR7/8 Agonists

Because thiol-binding mediates intratumoral retention, it should prevent the documented toxicity associated with the systemic exposure to TLR7 agonists.^16,47^ We observed that p(Man-TLR7-PDS) administered subcutaneously in healthy mice and intratumorally in B16F10 melanoma- and CT26 adenocarcinoma-bearing mice did not lead to weight loss (Figures S14–S16). To further characterize the systemic toxicity, we selected 3M-052 (telratolimod), which is a lipid-modified TLR7/8 agonist designed to form depots for controlled release from the injection site (or for incorporation into liposomes or cell membranes), as a benchmark for TLR7 agonist-induced inflammation.^48^ We intratumorally injected MC38 colon carcinoma-bearing mice with either p(Man-TLR7-PDS) or 3M-052 on a TLR7 equimolar basis and evaluated the production of systemic inflammatory cytokines. Here, we showed that 3M-052, but not p(Man-TLR7-PDS), produced significant upregulation of interferon gamma (IFN-γ), interleukin 6 (IL-6), monocyte chemoattractant protein 1 (MCP-1), interferon beta (IFN-β), and interleukin 27 (IL-27) as compared to PBS or vehicle control (Figures 5c–g and S17). This provides evidence that the systemic toxicity associated with other clinically relevant localized/controlled-release formulations is limited with p(Man-TLR7-PDS) therapy. We validated this result with a similar experiment in B16F10 melanoma, where we compared p(Man-TLR7-PDS) to nonbinding p(Man-TLR7) and observed a trend toward reduced cytokine levels (Figure S18).

## Discussion

In this work, we exploited redox imbalance in tumors to create an *in situ* cancer vaccine, in which a multifunctional polymer is conjugated to the tumor cell surface and tumor debris via disulfide exchange with exofacial unpaired cysteines, thus adjuvating tumor neoantigens. The polymer is endowed with TLR7 agonizing moieties and with mannose moieties to mark tumor debris for APC endocytosis and to further localize the TLR7 agonist within the endosome, the compartment in which the receptor is active.

By exploiting the fundamental dysregulated metabolic profile of solid tumors, our thiol-binding platform can be applied to virtually all solid tumor types in a tumor-agnostic manner. Similar chemistry has previously been employed to develop efficient MRI contrast agents, but therapeutic application has been limited.^49,50^ Importantly, this strategy is clinically relevant as the phenomenon of excessive oxidative stress and redox imbalance is also observed in human cancers,^32,51,52^ so we expect that PDS-mediated intratumoral cell binding and retention has high translational potential. We are also encouraged by the modularity of the platform. Other functional monomers can be incorporated, and larger agonists can be conjugated to the azide-terminated chain end (which was otherwise used only for conjugating a fluorophore here). With these approaches, we can use this platform to deliver different adjuvants or create combination therapies with potentially synergistic effects.^53^

Other common synthetic anticancer drug delivery strategies can be broadly grouped into two categories. The first are nanocarriers, which traditionally exploit the enhanced permeability and retention (EPR) effect, whereby disordered vasculature in the tumor allows for nanoscale materials to exit circulation, where reduced lymphatic drainage enables their retention. While this effect is strong in small animals, translation to humans has been limited due to accumulation in other organs.^54^ Current nanomaterial efforts focus on more specific targeting to the tumor.^30^ Our cysteine-reactive platform does not rely on passive accumulation but rather on local retention after intratumoral administration and does not require any specific targeting moieties, such as antitumor antibodies. The other category is intratumoral drug depots, where an injection forms a depot *in situ* to slowly release a drug over time.^55,56^ Our platform does not have a temporal-release aspect that requires very specific engineering. It further allows for covalent association of the drug with tumor cell surfaces rather than nonspecific local release, which in this example of an *in situ* vaccine may be particularly important.

Clinical translation of TLR7/8 agonists for cancer immunotherapy is an active area of research,^15^ with at least 10 ongoing clinical trials evaluating their efficacy in a variety of tumor types, many of which show promising early results (clinicaltrials.gov). There is already an FDA-approved TLR7/8 agonist, imiquimod, which is applied topically for superficial basal cell carcinoma.^57^ A variety of drug delivery strategies are under investigation to increase the therapeutic efficacy of these agonists.^17,58^ Our PDS-mediated tumor cell-surface-binding and tumor retention platform could further improve the therapeutic efficacy and/or toxicity profile of such therapeutics. It is important to note that our engineered polymeric construct, p(Man-TLR7-PDS), contains a TLR7 agonist, which is more relevant in murine models.^59^ However, we could envision instead the incorporation of a structurally similar dual TLR7/8 agonist monomer with activity on the human receptors (TLR8 is less relevant in murine models^60^).

Another promising delivery strategy for TLR7/8 agonists is through their conjugation to tumor-targeting antibodies such as trastuzumab, which binds HER2.^61^ While preclinical data is quite promising, major limitations exist, such as downregulation of tumor-specific targeting antigens^62,63^ and induction of antidrug antibodies,^64^ limiting their long-term clinical potential. Our delivery strategy circumvents these limitations by targeting a fundamental metabolic phenomenon rather than a tumor-specific antigen and by removing the potentially immunogenic protein component.

Intratumoral injection is becoming an increasingly viable administration route for cancer immunotherapies. While the medical community has traditionally favored systemic therapies, there are now over 300 ongoing clinical trials evaluating intratumorally injected therapeutics (clinicaltrials.gov). Recent improvements to surgical techniques have rendered a larger majority of cancers accessible for intratumoral injection.^65^ While the relevance of the abscopal effect in humans is still being investigated, we believe there is promise not only in the treatment of primary tumors but also in treating accessible metastases, in combination with systemic therapies such as checkpoint inhibitors to induce systemic antitumor immunity.^66,67^

In conclusion, our drug delivery platform is promising for three main reasons. First is its simplicity—we used well-studied thiol-reactive chemistry to exploit a fundamental metabolic feature of solid tumors. The second is its modularity—the immune-active monomers can be substituted with other small molecule agonists, and the chain end can be conjugated to larger adjuvant molecules. Finally, our polymer is translationally relevant—other TLR7/8 agonist formulations are in development and show significant clinical promise. The demonstration we provide here for *in situ* cancer vaccines shows efficacy in both monotherapy and in combination with checkpoint inhibition and reduction of systemic toxicity of the immune agonist compared to another slow release formulation.

## Materials and Methods

### Mice and Cancer Cell Lines

Female C57BL/6 mice (aged 8–12 weeks) were purchased from Charles River Laboratory. Female Balb/c mice (aged 8–12 weeks) were purchased from Jackson Laboratory. B16F10 murine melanoma, CT26 and MC38 murine colon carcinoma, EMT6 murine mammary carcinoma, and MDA-MB-231 human breast adenocarcinoma cell lines were purchased from ATCC and cultured according to instructions, with routine checks for mycoplasma contamination. Tumor inoculations were 500 000 cells in 30 μL of sterile PBS unless otherwise noted. Polymer solutions were verified as endotoxin-free prior to injection via HEK-Blue TLR4 reporter cells (InvivoGen). Tumor dimensions were measured with digital calipers, and volume was calculated as height × width × thickness × (π/6). All of the animal experiments performed in this research were approved by the Institutional Animal Care and Use Committee of the University of Chicago under protocol 72456.

### PET-RAFT Polymerization

Briefly, monomers and chain transfer agent (CTA) were dissolved in 1 mL of DMSO in a Schlenk tube. Eosin Y was added at 0.02 equiv to CTA in 50 μL of DMSO. The tube was sealed and degassed via four freeze-pump thaw cycles then placed inside a foil-wrapped bowl with green LED strip lights. The reaction was covered with foil and left stirring for 14 h. After that time, the polymer was precipitated in cold diethyl ether three times to remove residual monomer. The resulting polymer was dried under reduced pressure and characterized with ^1^H NMR and GPC using InfinityLab EasiVial PMMA standards (Agilent, cat. no. PL2020–0202). Prior to *in vivo* administration, polymers were dissolved in sterile water and purified using 7kD MWCO Zeba desalting columns (Thermo Scientific, cat. no. 89883). The process is described in detail with monomer masses used in the Supplemental Methods.

### *In Vitro* Cancer Cell Binding of p(PDS)

For detection, AZDye 647 DBCO (Click Chemistry Tools, cat. no. 1302–25) was conjugated to polymer via the azide chain end (leading to 1:1 conjugation), and unconjugated dye was removed using Zeba desalting columns with a 7K MWCO (Thermo Scientific, cat. no. 89883). Cells were removed from culture, washed free of media with PBS two times, and then incubated on ice with various concentrations of dye labeled polymer for 90 min. For the pretreatment experiment, cells were treated with 500 μM N-ethyl maleimide (NEM, Sigma-Aldrich, cat. no. 04259) or tris(2-carboxyethyl)phosphine (TCEP, Sigma-Aldrich, cat. no. 75259) for 30 min and washed twice with PBS prior to polymer incubation. After polymer incubation, cells were washed twice with PBS and resuspended in 2% v/v heat-inactivated fetal bovine serum (FBS, ThermoFisher, cat. no. A3840002) in PBS for flow cytometric analysis. Cells were acquired on BD LSRFortessa, and data were analyzed via FlowJo. Polymer (APC-A) mean fluorescence intensity (MFI) of singlet cells was recorded and compared between groups.

### Histological Analysis of Tumor Retention

B16F10 tumor-bearing mice were injected intratumorally with 30 μL of dye labeled polymer at a concentration of 200 μM fluorophore once tumors reached an average size of ∼80 mm^3^. Six hours after injection, tumors were collected and embedded in OCT compound (ThermoFisher, cat. no. 23730571) and frozen at −80 °C before sectioning on a microtome-cryostat into 5 μm sections. The slides were then stained with AlexaFluor 594 conjugated antithioredoxin-1 (polyclonal, Novus Biologicals, cat. no. 89458AF594). Slides were then fixed with ProLong Gold antifade reagent with DAPI (Fisher Scientific, cat. no. P36931) and imaged with Olympus IX83 spinning-disc confocal fluorescence microscope (Olympus, Tokyo, Japan).

### Flow Cytometric Analysis of Cell Binding *In Vivo*

B16F10 tumors were inoculated intradermally into the backs of 8-week-old female C57BL/6 mice as described previously. Once tumors reached an average size of ∼100 mm^3^, 30 μL of AZ647 dye labeled polymer (for Figure 2: molecular weight-matched, containing high or low weight fraction PDS, or none for nonbinding control; for Figure 3: p(Man-TLR7-PDS) or p(Man-TLR7), as used in efficacy studies) was injected intratumorally at a concentration of 200 μM fluorophore. Three hours after injection, tumors were collected and digested for 30 min at 37 °C. Digestion medium was pyruvate free DMEM (Gibco, ThermoFisher, cat. no. 10313021) supplemented with 5% FBS, 3.3 mg/mL collagenase D (Sigma-Aldrich, cat. no. 11088858001), 1 mg/mL collagenase IV (Worthington Biochemical, Fisher Scientific, cat. no. NC9919937), and 1.2 mM CaCl_2_. Single-cell suspensions were prepared using a Corning Falcon 70 μm cell strainer (Fisher Scientific, cat. no. 08–771–2). Red blood cells were lysed with 3 mL of ACK lysing buffer (Gibco, ThermoFisher, cat. no. A10492–1) for 90 s and neutralized with 15 mLof DMEM supplemented with 5% FBS. Cell viability was determined using LIVE/DEAD Fixable Violet Dead Cell Stain (405 nm excitation, Invitrogen, ThermoFisher, cat. no. L34955). Staining with PE antimouse CD45 (clone 30-F11, BioLegend, cat. no. 103106) and Alexa Fluor 488 anti-TRP1 (clone EPR21960, Abcam, cat. no. ab270104) was done in 2% FBS in PBS. Cells were acquired on BD LSRFortessa, and data was analyzed via FlowJo. Polymer MFI and frequency of polymer positive cells of each population and polymer treatment were measured and compared.

### *In Vitro* Activity of p(Man-TLR7-PDS)

RAW 264.7 macrophage-like cells were purchased from ATCC and cultured according to instructions. One day after plating in a flat-bottom, nontreated 96 well plate, cells were treated with various concentrations of TLR7 equiv polymer or R848 (Sigma-Aldrich, cat. no. SML0196) (as quantified by absorbance at 327 nm). Supernatant was collected 24 h after treatment and analyzed via LEGENDplex Mouse Inflammation Panel (BioLegend, cat. no. 740446).

### Antitumor Efficacy of p(Man-TLR7-PDS) vs Nonbinding p(Man-TLR7)

For the melanoma model, B16F10 cells were inoculated intradermally in the shaved left shoulder of 8-week-old female C57BL/6 mice. Mice bearing established tumors were injected intratumorally with 40 μg of TLR7 equiv (as quantified by absorbance at 327 nm, based on the equation: TLR7 content = 1.9663 * *A*_372_ + 0.0517) of either p(Man-TLR7-PDS) or p(Man-TLR7) in 30 μL of sterile PBS or vehicle only control on days 6, 9, and 12 after tumor inoculation. The volume of the tumor was recorded as previously described. Mice were euthanized when the tumor volume exceeded 500 mm^3^ and/or based on humane end-point criteria. For the mammary carcinoma model, EMT6 cells were inoculated into the left mammary fat pad of 8-week-old female BALB/c mice. Mice bearing established tumors were injected intratumorally with 40 μg of TLR7 equiv of either p(Man-TLR7-PDS) or p(Man-TLR7) in 30 μL of sterile PBS or vehicle only control on days 6, 9, 12, and 15 after tumor inoculation. The volume of the tumor was recorded as described above. Mice were euthanized when the tumor volume exceeded 750 mm^3^ and/or based on humane end-point criteria.

### Antitumor Efficacy of p(Man-TLR7-PDS) in Combination with CPI

8-week-old female Balb/c mice were inoculated with CT26 cells subcutaneously as previously described. On days 6, 9, 12, and 15 after tumor inoculation, mice were injected intratumorally with 40 μg of TLR7 equiv p(Man-TLR7-PDS) in 30 μL of sterile PBS or vehicle only control and intraperitoneally with 100 μg of anti-PD-1 (29F.1A12, BioXCell, cat. no. BE0273) in 100 μL of sterile PBS. Tumor growth was recorded as described above, and mice were euthanized when the tumor volume exceeded 750 mm^3^ and/or based on humane end-point criteria.

### Dose-Dependent Efficacy of p(Man-TLR7-PDS) in MC38 Colon Carcinoma

8-week-old female C57BL/6 mice were inoculated with MC38 colon carcinoma cells subcutaneously as described above. On days 6, 9, and 12 after tumor inoculation, mice were injected intratumorally with 20 or 40 μg of TLR7 equiv p(Man-TLR7-PDS) in 30 μL of sterile PBS or vehicle-only control. Tumor growth was recorded as described above, and mice were euthanized when the tumor volume exceeded 500 mm^3^ and/or based on humane end-point criteria.

### Efficacy of p(Man-TLR7-PDS) with Cellular Depletions

8-week-old female C57BL/6 mice were inoculated with MC38 colon carcinoma cells subcutaneously as described above. Depletion antibodies were administered starting 1 day before treatment, which was started when tumors reached an average size of ∼100 mm^3^ and were discontinued after treatment. CD4 (clone GK1.5, BioXCell, cat. no. BE0003–1) and CD8α (Clone 2.43 BioXCell, cat. no. BE0061) depletion antibodies and isotype control (BioXCell, cat. no. BE0086) were administered at a dose of 400 μg every 3 days. CSF1R (clone ASF98, BioXCell, cat. no. BE0213) depletion antibodies were administered at a dose of 300 μg every other day. Treatment with p(Man-TLR7-PDS), 40 μg of TLR7 equiv in 30 μL of sterile PBS, occurred every 3 days for a total of three doses. Tumor growth was recorded as described above, and mice were euthanized when the tumor volume exceeded 500 mm^3^ and/or based on humane end-point criteria.

### Serum Cytokine Analysis

Mice bearing established MC38 tumors were injected intratumorally with 40 μg of TLR7 equivalent (as quantified by absorbance at 273 nm, relative to a standard curve) polymer (full or nonbinding control) in 30 μL of sterile PBS. As a comparison, other mice were injected with TLR7/8 agonist telratolimod (3M-052, MedChemExpress, cat. no. HY-109104) in 10% DMSO, 40% PEG300 (Sigma-Aldrich, cat. no. 8.07484), and 5% Tween-80 in PBS (Sigma-Aldrich, cat. no. P8074) (with additional vehicle control mice). Six hours after injection, plasma was collected via submandibular bleed and analyzed for proinflammatory cytokines using LEGENDplex Mouse Inflammation Panel Assay (BioLegend, cat. no. 740446).

### Statistical Analysis

Statistical analysis was performed using Prism (v8, GraphPad Prism). For multiple comparisons, one-way analysis of variance followed by a Tukey post hoc test was used. For direct comparisons, an unpaired *t*-test was used. For survival, pairwise log-rank (Mantel-Cox) tests were used.

## Notes

Additional data are available upon request.

## Supporting Information Available

Additional figures and methods as described in the text (PDF)
