# Supplementary material for: Tumor Cell-Surface Binding of Immune Stimulating Polymeric Glyco-Adjuvant via Cysteine-Reactive Pyridyl Disulfide Promotes Antitumor Immunity
Source: ACS Cent Sci. 2022 Oct 7;8(10):1435–46. doi: 10.1021/acscentsci.2c00704 (PMC9615125; doi:10.1021/acscentsci.2c00704)
Supplement: Supplementary file 1 — oc2c00704_si_001.pdf [file oc2c00704_si_001.pdf]

Tumor cell-surface binding of immune stimulating polymeric glyco-adjuvant via cysteine-reactive pyridyl disulfide promotes antitumor immunity

Authors
Anna J. Slezak[1], Aslan Mansurov[1], Michal M. Raczy[1], Kevin Chang[1], Aaron T. Alpar[1], Abigail L. Lauterbach[1], Rachel P. Wallace[1], Rachel K. Weathered[1], Jorge E.G. Medellin[1], Claudia Battistella[1], Laura T. Gray[1], Tiffany M. Marchell[2], Suzana Gomes[1], Melody A. Swartz[1,2,3,4], Jeffrey A. Hubbell[1,2,4]*.
*Corresponding author. Email: jhubbell@uchicago.edu

Affiliations
[1]Pritzker School of Molecular Engineering, University of Chicago, Chicago, IL, USA.
[2]Committee on Immunology, University of Chicago, Chicago, IL, USA.
[3]Ben May Department for Cancer Research, University of Chicago, Chicago, IL, USA.
[4]Committee on Cancer Biology, University of Chicago, Chicago, IL, USA.

Contents: **Page**

Supplementary Figures S1-S19 S1-S13
Supplementary Methods S14-S22

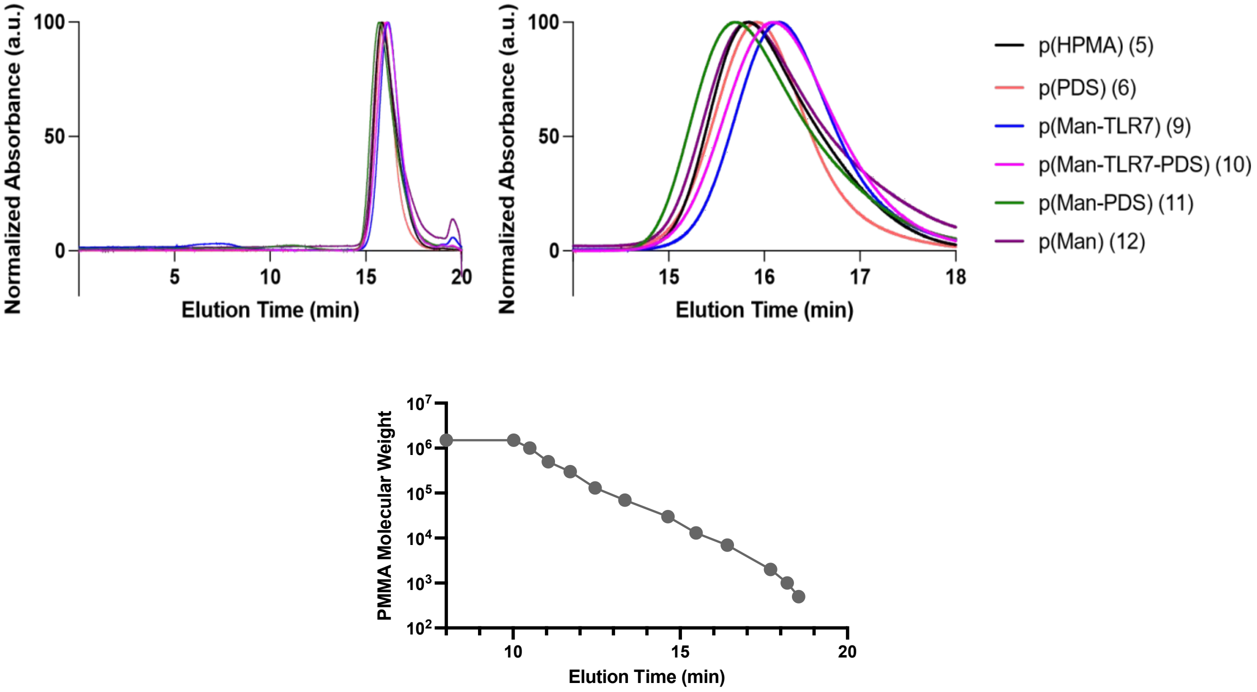

Fig. S1. GPC elution profiles of all synthesized polymers. Gel permeation chromatography was performed using Tosoh EcoSEC size exclusion chromatography system with Tosoh SuperAW3000 + Tosoh SuperAW4000 columns, eluted in DMF + 0.01 M LiBr at 50°C. Full elution profile and magnification of relevant region shown, along with the PMMA calibration standards.

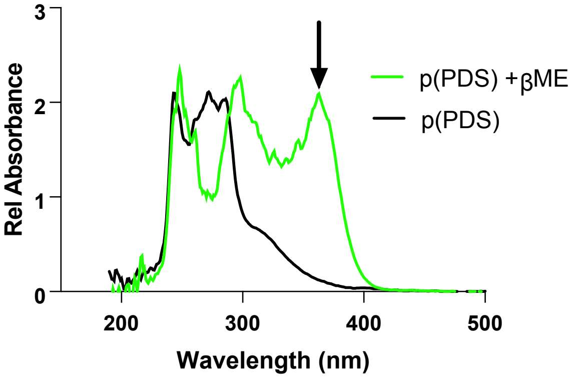

Fig. S2. Extent of PDS reduction can be monitored with UV-Vis Spectrophotometry. p(PDS) was dissolved in PBS and the full UV-Vis absorbance spectrum was recorded. Reducing

agent β-mercaptoethanol was added in 100x excess of PDS and allowed to react to completion (30 minutes). This verifies that the PDS groups are functionally active and can be used to quantify PDS content using a standard curve of 2-mercaptopyridine.

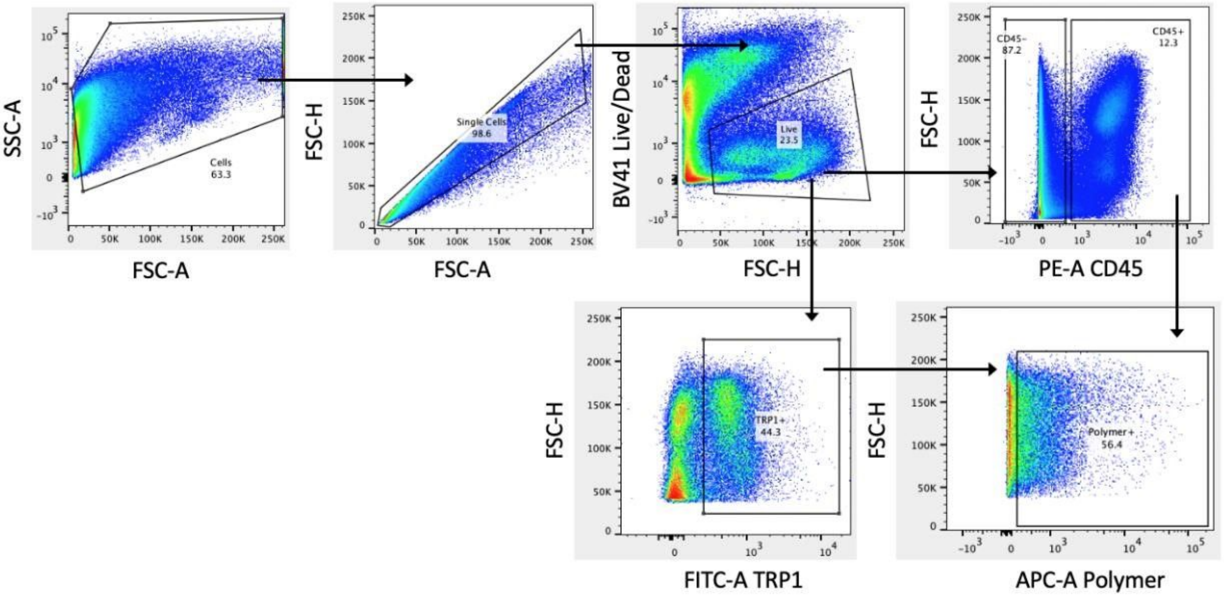

Fig. S3. Gating strategy for *in vivo* binding of PDS polymers. Cells were collected on BS LSRFortessa and data were analyzed in FlowJo. Cells were gated on FSC-A vs. SSC-A, single cells were gates on FSC-A vs. FSC-H, and live cells were gated on FSC-H vs. BV-421. From there, $CD45^{+}$ cells were gated on PE-A vs. FSC-H. Alternatively, $TRP1^{+}$ cells were gated on FITC-A vs. FSC-H. The positive populations from each of those gates was gated for $Polymer^{+}$ cells on APC-A vs. FSC-H. Alternatively, the APC MFI of the whole $TRP1^{+}$ or $CD45^{+}$ populations was recorded.

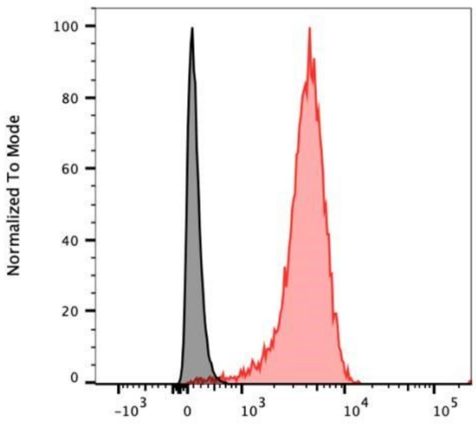

Fig. S4. Intracellular thioredoxin-1 is detectable in B16F10 cells via flow cytometry. B16F10 cells were removed from culture, washed free of media with PBS two times, then permeabilized using FIX&PERM Cell Permeabilization Kit (ThermoFisher) for intracellular antibody staining with AF594-conjugated anti-thioredoxin-1 (Novus Biologicals). Cells were collected by flow cytometry as described in methods. Representative histogram of staining shown.

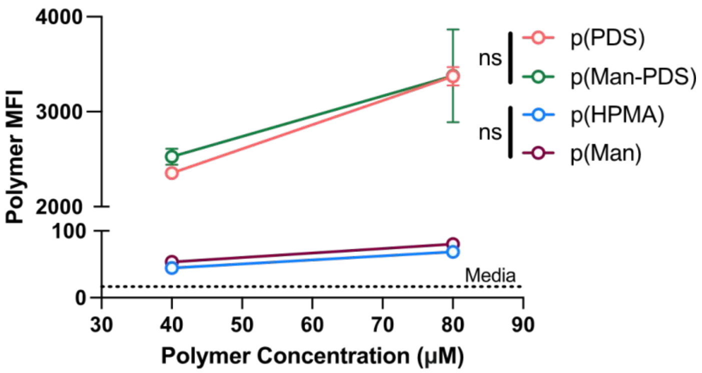

Fig. S5. Polymer binding to tumor cells is not mannose dependent. EMT6 cells were removed from culture, washed free of media with PBS two times, then incubated on ice with various concentrations of AZDye 674-labeled polymer for 90 minutes (n = 3, mean +/- SEM). Polymers had similar numbers of mannose monomer or PDS monomer per chain as quantified by proton NMR and were molecular weight matched. Cells were collected by flow cytometry as

described in methods, where MFI correlates to amount of polymer attached to the cells. Polymers containing PDS monomers showed concentration dependent binding to tumor cells, regardless of the incorporation of mannose monomer. Likewise, polymers without PDS did not show significant binding to tumor cells, regardless of mannose monomer. Statistical analyses were performed with ordinary one-way analysis of variance with Tukey's test.

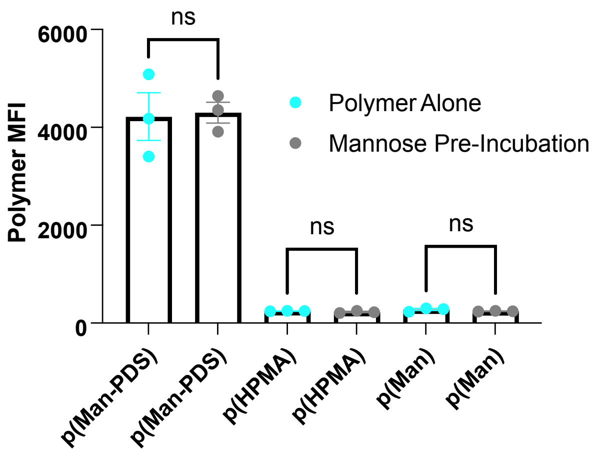

Fig. S6. PDS polymer binding to tumor cells is not altered by mannose pre-incubation. B16F10 cells were removed from culture, washed free of media with PBS two times, then incubated with 1 mg/mL D-mannose on ice for 30 minutes. AZDye 674-labeled polymer was added directly to the mannose mixture to make a polymer solution at 80 μM fluorophore then incubated for an additional 90 minutes ($n = 3$, mean +/- SEM). Cells were collected by flow cytometry as described in methods, where MFI correlates to amount of polymer attached to the cells. Binding is confirmed to be independent of mannose pre-incubation, as the polymer MFI does not change. Statistical differences were determined by unpaired t-tests.

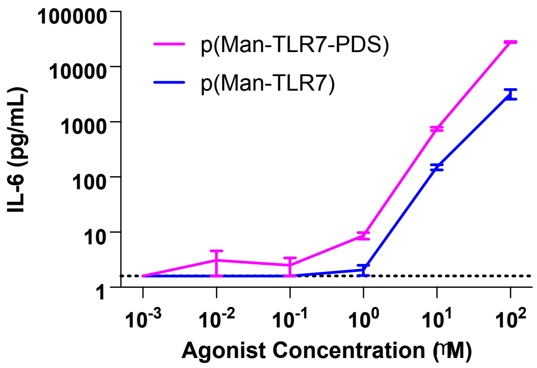

Fig. S7. p(Man-TLR7-PDS) stimulates murine BMDCs. Murine bone marrow-derived dendritic cells (BMDCs) were treated with various concentrations of p(Man-TLR7-PDS) or p(Man-TLR7) on a TLR7 monomer basis. Supernatant IL-6 was measured after 6 hours via LEGENDplex (n = 3, mean +/- SEM). Results show that both the binding and non-binding polymer variants can stimulate dendritic to produce pro-inflammatory cytokines.

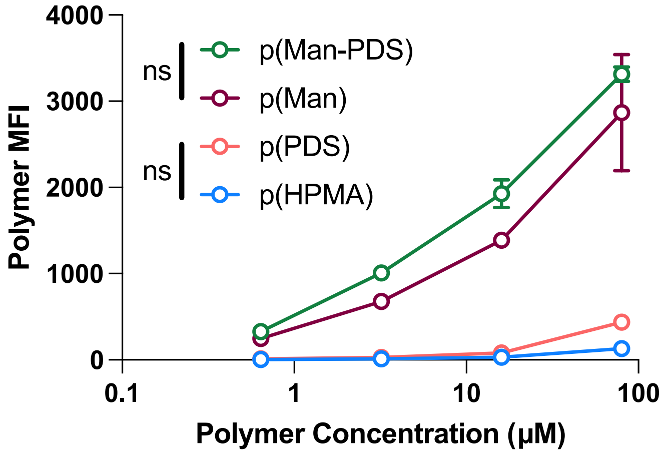

Fig. S8. Polymer uptake by BMDCs is mediated by mannose. Murine bone marrow-derived dendritic cells (BMDCs) were incubated with various concentrations of AZ647 dye-labelled polymer in complete Lutz media for 45 minutes. The cells were washed and fixed with 2% paraformaldehyde in PBS for flow cytometric analysis. Cells were collected by flow cytometry as described in methods, where MFI correlates to amount of polymer taken up by the cells.

Uptake is confirmed to be dependent on the incorporation of mannose and is not significantly affected by PDS-mediated binding. Statistical differences were determined by unpaired t-tests.

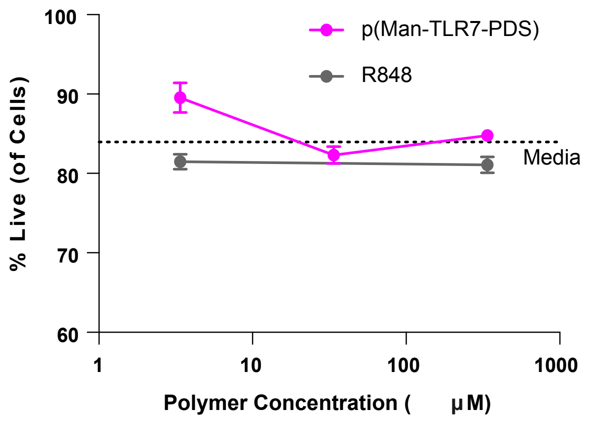

Fig. S9. p(Man-TLR7-PDS) is not inherently cytotoxic to RAW 264.7 macrophages. RAW 264.7 macrophage-like cells were purchased from ATCC and cultured according to instructions. One day after plating in a flat-bottom, non-treated 96 well plate, cells were treated with various concentrations of TLR7 equivalent polymer (as quantified by absorbance at 327 nm). 24 hours after treatment, cells were stained with Zombie Aqua Fixable Viability Dye (BioLegend) and collected by flow cytometry as described in methods. Live cells as a percent of total cells is shown at various polymer or control TLR7/8 agonist R848 (Sigma), with no significant change from the media alone control wells.

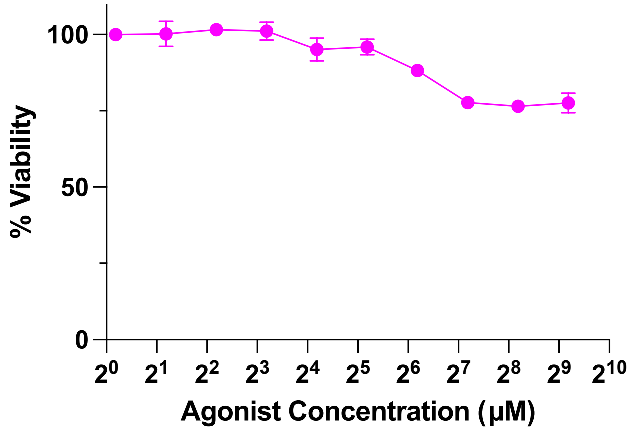

Fig. S10. p(Man-TLR7-PDS) is not inherently cytotoxic to B16F10 melanoma cells. Cells were seeded at a density of 8,000 cells per well and incubated overnight with various concentrations of p(Man-TLR7-PDS). The following day, the cells were analyzed using MTT Cell Viability Assay (ThermoFisher) according to manufacturer's protocol. Even at high concentrations of polymer, high cell viability was observed.

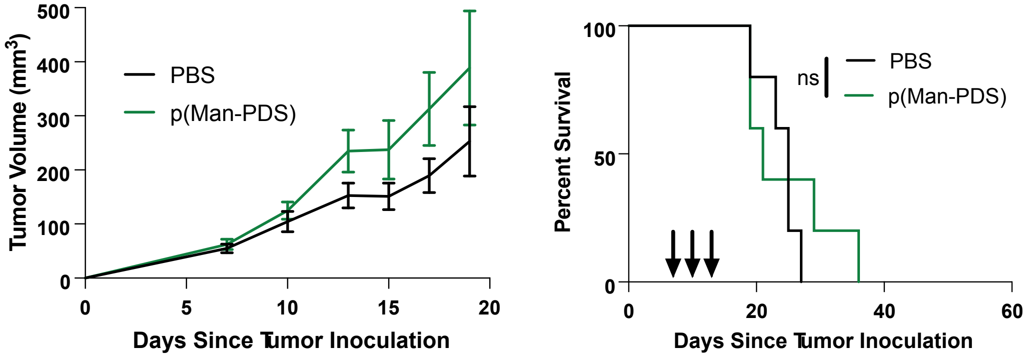

Fig. S11. p(Man-PDS) has no inherent antitumor efficacy. 8-week-old female C57BL/6 mice were inoculated with MC38 tumors as described in methods. On days 7, 10, and 13 postinoculation, mice were injected intratumorally with 260 µg p(Man-PDS), which corresponds by total mass to our typical dose of 40 µg TLR7 monomer (*n* = 6, mean +/- SEM). The volume of the tumor was recorded as previously described. Mice were euthanized when the tumor

volume exceeded 500 mm^3 and/or based on humane end-point criteria. Here we demonstrate that the non-adjuvanted p(Man-PDS) has no inherent antitumor efficacy. Statistical differences were determined by pairwise log-rank (Mantel-Cox) tests.

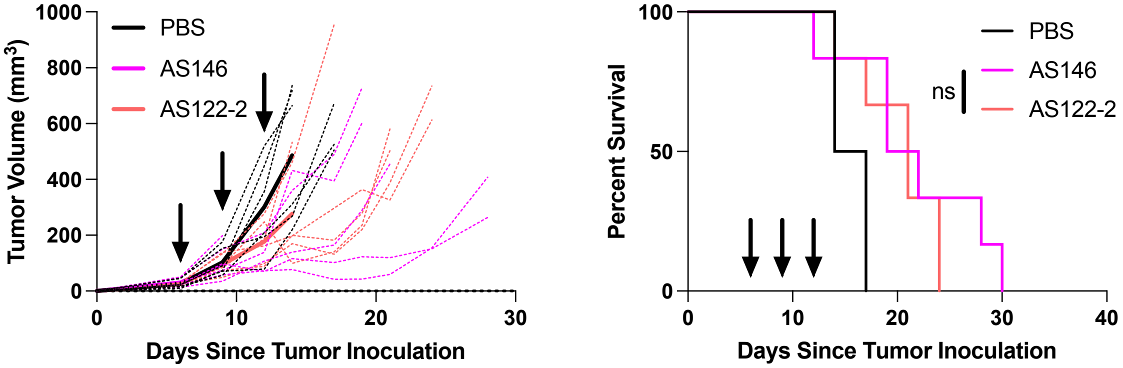

Fig. S12. p(Man-TLR7-PDS) has low batch-to-batch variability. Two batches of p(Man-TLR7-PDS), AS146 and AS122-2, were synthesized using separate batches of CTA, mannose monomer, PDS monomer, and TLR7 monomer. 8-week-old female C57BL/6 mice were inoculated with B16F10 tumors as described in methods. On days 6, 9, and 12 post-inoculation, mice were injected intratumorally with 40 µg TLR7 monomer equivalent of AS146 or AS122-2 (n = 7) or vehicle control (PBS, n = 6). The volume of the tumor was recorded as previously described. Mice were euthanized when the tumor volume exceeded 500 mm^3 and/or based on humane end-point criteria. Here we verify the low variability of batches of p(Man-TLR7-PDS).

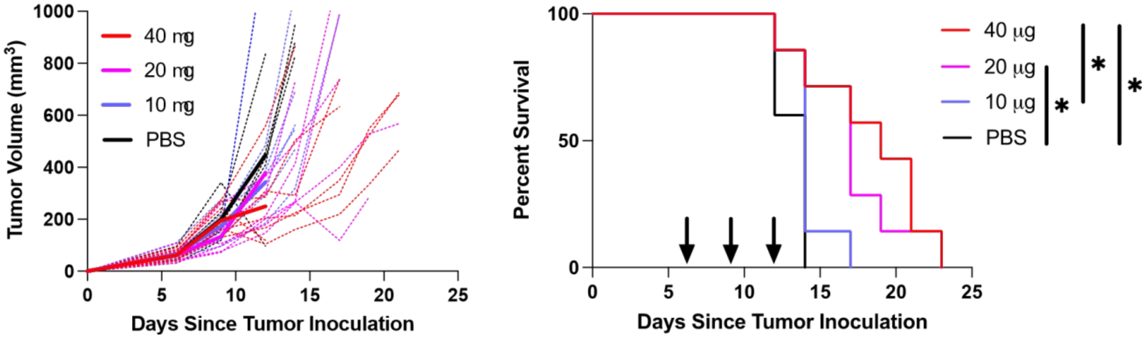

Fig. S13. Dose dependent effect of p(Man-TLR7-PDS) in B16F10 melanoma. 8-week-old female C57BL/6 mice were inoculated with B16F10 tumors as described in methods. On days 6, 9, and 12 post-inoculation, mice were injected intratumorally with various concentrations of polymer (*n* = 7) or vehicle control (PBS, *n* = 5) (mean +/- SEM). The volume of the tumor was recorded as previously described. Mice were euthanized when the tumor volume exceeded 500 mm^3 and/or based on humane end-point criteria. Here we demonstrate that the polymer has dose-dependent antitumor efficacy and significantly prolongs survival of tumor-bearing mice. Statistical differences were determined by pairwise log-rank (Mantel-Cox) tests. *p < 0.05.

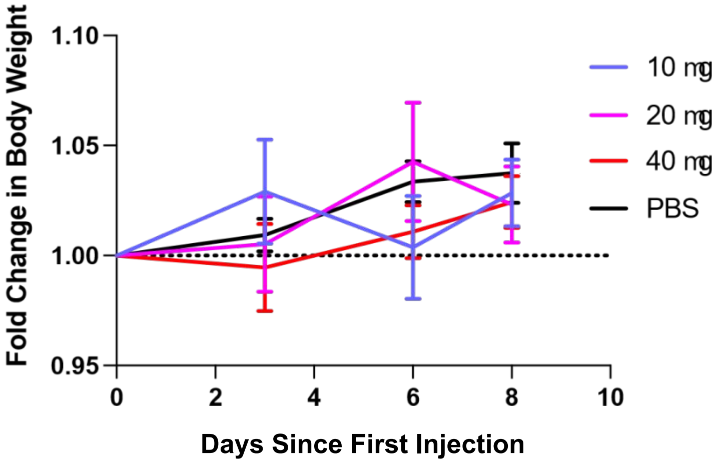

Fig. S14. p(Man-TLR7-PDS) does not cause weight loss in healthy mice. Healthy 8-week-old female C57BL/6 mice were injected subcutaneously three times three days apart with various

concentrations of polymer in sterile PBS (or vehicle control) (n = 5, mean +/- SEM). Doses were defined by TLR7 monomer content as quantified by absorbance at 327 nm, based on a standard curve of monomer. Mouse weight was recorded on each injection day and two days after the final injection. At all doses and timepoints, mice did not, on average, lose a significant portion of body weight.

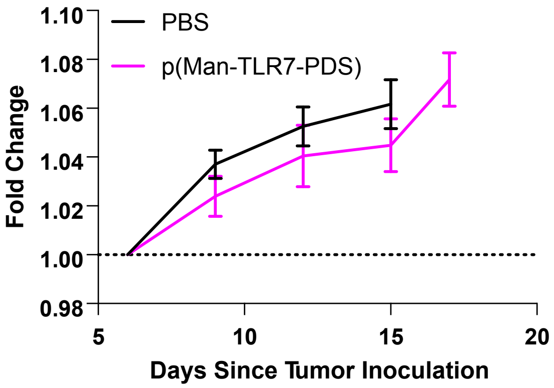

Fig. S15. p(Man-TLR7-PDS) does not cause weight loss in tumor-bearing BALB/c mice. 8week-old female BALB/c mice were inoculated with CT26 tumors as described in methods. On days 6, 9, and 12 post-inoculation, mice were injected intratumorally with 40 µg TLR7 monomer equivalent p(Man-TLR7-PDS) (n = 8) or vehicle control (PBS, n = 7) (mean +/- SEM). The mouse weights were recorded every three days. Mice were euthanized when the tumor volume exceeded 500 mm^3 and/or based on humane end-point criteria.

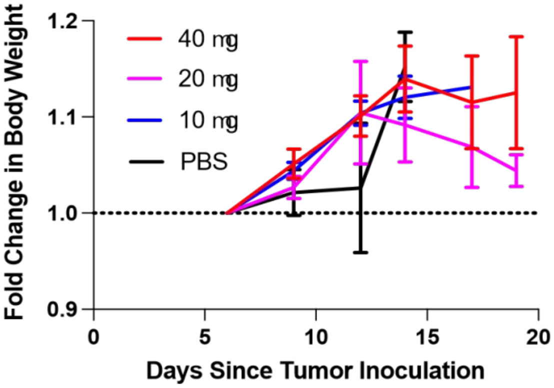

Fig. S16. p(Man-TLR7-PDS) does not cause weight loss in tumor-bearing C57BL/6 mice. 8week-old female C57BL/6 mice were inoculated with B16F10 tumors as described in methods. On days 6, 9, and 12 post-inoculation, mice were injected intratumorally with various concentrations of p(Man-TLR7-PDS) (n = 7, mean +/- SEM). The mouse weights were recorded every three days. Mice were euthanized when the tumor volume exceeded 500 mm^3 and/or based on humane end-point criteria.

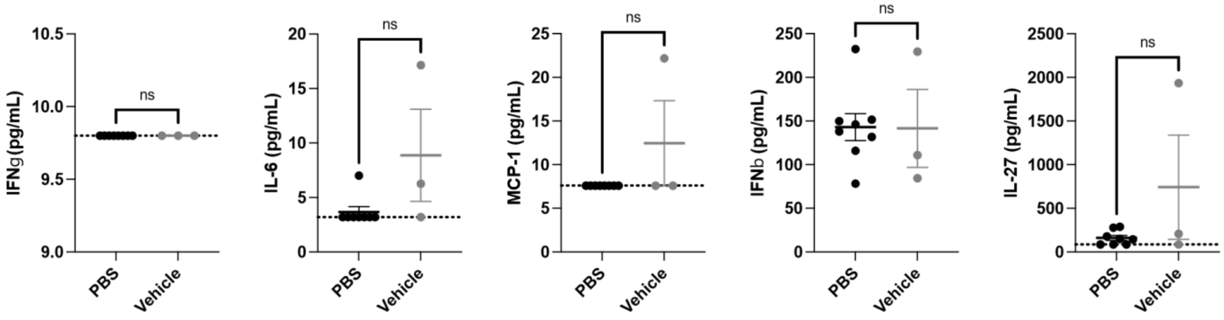

Fig. S17. Vehicle for 3M-052 injection does not produce systemic cytokines compared to PBS. 8-week-old female C57BL/6 mice were inoculated with MC38 tumors as described in methods. Mice were injected intratumorally with 30 µL PBS (n = 8) or vehicle control consisting of 10% DMSO 40% PEG300, and 5% Tween-80 in PBS (n = 3). Six hours after injection, sera was collected and analyzed for proinflammatory cytokines, including INFγ, IL-6, MCP-1, IFNβ, and IL-27 (mean +/- SEM). Statistical analyses were performed using Mann-Whitney tests for nonparametric data.

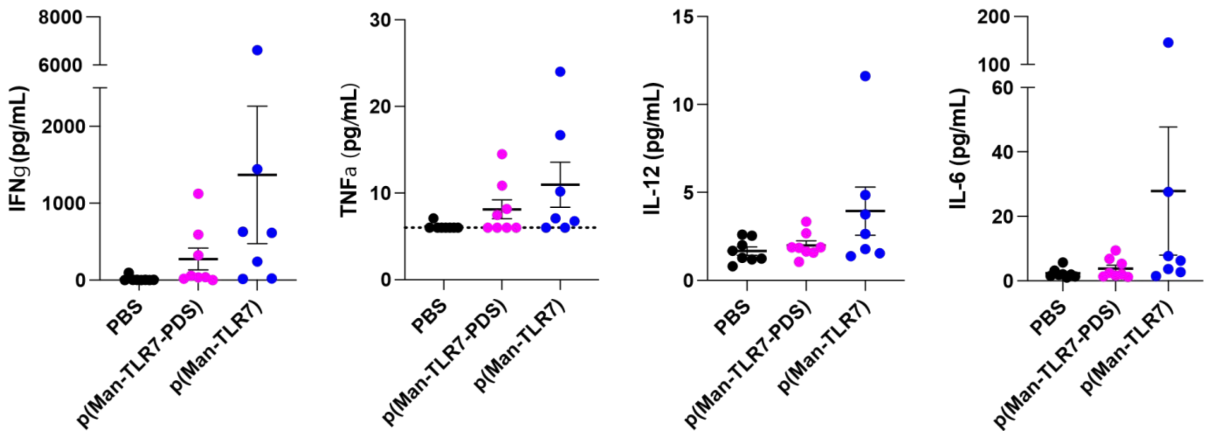

Fig. S18. Intratumorally injected p(Man-TLR7-PDS) does not induce upregulation of systemic proinflammatory cytokines. 8-week-old female C57BL/6 mice were inoculated with B16F10 tumors as described in methods. On days 6 and 9 post-inoculation, mice were injected intratumorally with 40 μg TLR.7 monomer equivalent p(Man-TLR7-PDS) or p(Man-TLR7) or vehicle control (PBS). On day 11, sera was collected and analyzed for proinflammatory cytokines, including INFγ, TNFα, IL-12p70, and IL-6 (n = 7 for all groups, mean +/- SEM). Statistical analyses were performed using Kruskal-Wallis tests followed by Dunn's multiple comparison for nonparametric data. All differences are non-significant.

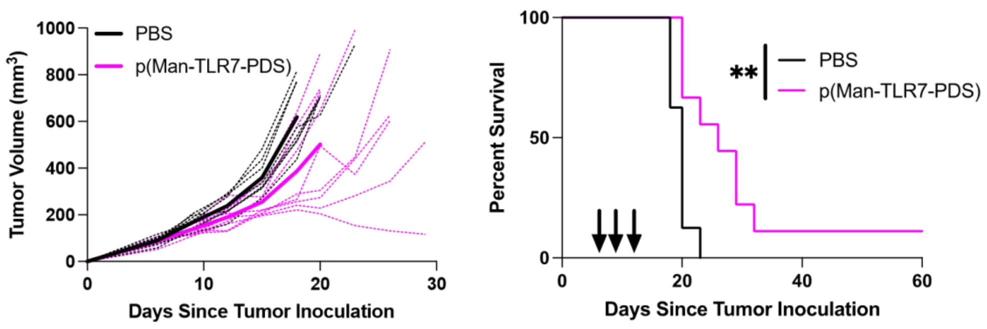

Fig. S19. p(Man-TLR7-PDS) has significant antitumor efficacy in ectopic EMT6. 8-week-old female BALB/c mice were inoculated with 500,000 EMT6 mammary carcinoma cells subcutaneously into the shaved left shoulder. On days 6, 9, and 12 post-inoculation, mice were

injected intratumorally with 40 μg TLR7 monomer equivalent p(Man-TLR7-PDS) (*n* = 8) or vehicle control (PBS, *n* = 7). The volume of the tumor was recorded as previously described. Mice were euthanized when the tumor volume exceeded 500 mm^3 and/or based on humane endpoint criteria. Shown are individual (thin dotted lines) and mean (thick solid lines) tumor growth curves and survival plot. Arrows denote treatment days. Statistical differences were determined by pairwise log-rank (Mantel-Cox) tests.

Supplementary Methods

No unexpected or unusually high safety hazards were encountered conducting this work.

Monomer and polymer synthesis

All chemicals were reagent grade and purchased from Sigma Aldrich and used as received unless otherwise noted. 2-(2-(2-(2-azidoethoxy)ethoxy)ethoxy) ethyl 4-cyano-4(((dodecylthio)carbonthioyl)thio)pentanoate (4), N-[2-(α-D-mannose)ethyl] methacrylamide (7), and N-(6-((4-((4-amino-2-(ethoxymethyl)-1H-imidazo[4,5-c]quinolin-1yl)methyl)benzyl)amino)hexyl) methacrylamide (8) were synthesized as previously described by Wilson et al. (2019). All other protocols listed in the following pages. All NMR spectra were collected on a Bruker Avance III HD Nanobay 400 MHz NMR unless otherwise noted and NMR spectra were analyzed with MNova (MestreLab). Gel permeation chromatography was performed using Tosoh EcoSEC size exclusion chromatography system with Tosoh SuperAW3000 + Tosoh SuperAW4000 columns, eluted in DMF + 0.01 M LiBr at 50°C. Mass spectrometry analysis was performed on Agilent 6130 LCMS using positive mode direct flow inject analysis.

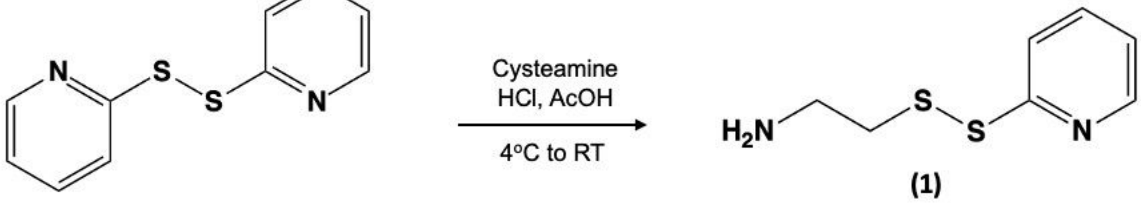

2-(pyridin-2-yldisulfaneyl)ethan-1-amine (1). 2,2'-Dipyridyldisulfide (4.4 g, 20 mmol) was dissolved in 20 mL methanol with 0.4 mL acetic acid (6.98 mmol) on ice with constant stirring. To that solution, cysteamine hydrochloride (1.2 g, 15.5 mmol) in 10 mL methanol was added dropwise over 15 min. After 24 hours, crude product was precipitated 4 times in cold diethyl ether then dried overnight under vacuum, providing 3.62 g (97.3%) of pure white powder. $C_7H_{10}N_2S_2$,, ESI-MS $[M+H]^+_{theor}$ = m/z 187.037, $[M+H]^+_{found}$ = 187.1 m/z . ^{1}H NMR (400 MHz, CD_3OD) δ 8.55 (d, 1H), 7.81 (m, 1H), 7.86 (d, 1H), 7.33 (m, 1H), 3.15 (t, 2H), 3.05 (t, 2H).

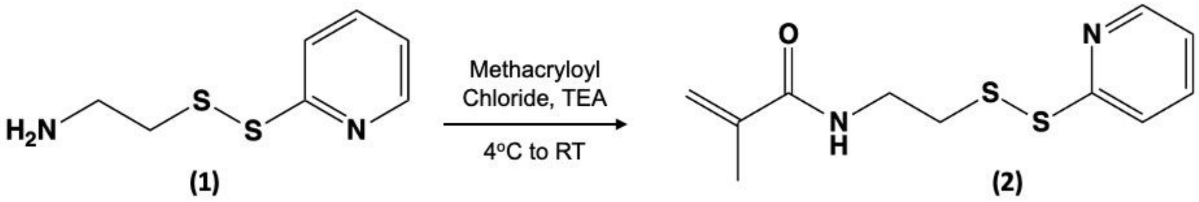

***N*-(2-(pyridine-2-yldisulfaneyl)ethyl)methacrylamide (PDS monomer) (2).** Compound **1** (2 g, 10.8 mmol) was dissolved in 10 mL DCM with 3 mL triethylamine (21.6 mmol) and stirred on ice. Methacryloyl chloride (1.35 g, 13.0 mmol) in 2 mL DCM was added dropwise. The reaction was left to react overnight, then extracted with water and DCM three times. The organic phase was dried over magnesium sulfate, filtered, and adsorbed on silica. The crude product was purified via flash column chromatography (Hex:EtOAc 1:2 v/v) to give 1.6 g (61.1%) of pure product: $C_{11}H_{14}N_2OS_2$, ESI-MS $[M+H]^+_{theor}$ = m/z 255.063, $[M+H]^+_{found}$ = 255.1 m/z . ^{1}H NMR (400 MHz,D_2O) δ 8.42 (d, 1H), 7.83 (q, 2H), 7.25 (d, 1H), 5.74 (s, 1H), 5.42 (s, 1H), 3.52 (q, 2H), 3.01 (t, 2H), 1.96 (s, 3H).

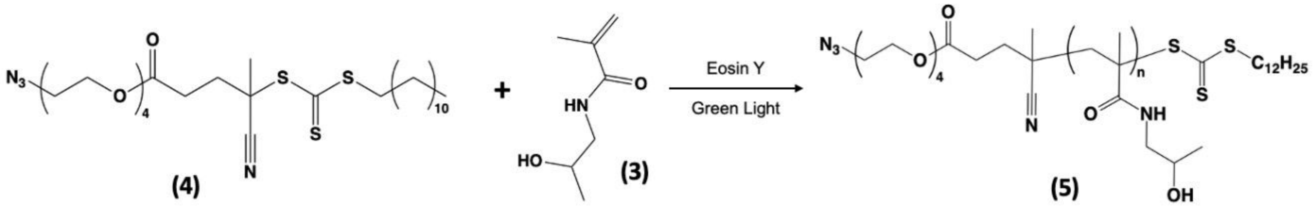

p(HPMA) (5). N-(2-Hydroxypropyl) methacrylamide (HPMA) **(3)** was recrystallized in acetone and dried prior to use. HPMA **(3)** (215 mg, 1.51 mmol) was dissolved in 1 mL DMSO with azide-functionalized trithiocarbonate chain transfer agent (CTA) **(4)** (13 mg, 0.0215 mmol) in a schlenk tube. Photo initiator Eosin Y (0.298 mg, 0.00043 mmol) was added in 50 μL DMSO. The tube was sealed and degassed via four freeze-pump thaw cycles then placed inside a foilwrapped bowl with green LED strip lights. The reaction was covered with foil and left stirring for 14 hours. After that time, the polymer was precipitated in cold diethyl ether three times to remove residual monomer. The resulting polymer (150 mg) was dried under reduced pressure and characterized with ^{1}H NMR and GPC using PMMA standards. The p(HPMA) used in binding studies had a number averaged molecular weight of 7,339 and a dispersity of 1.327. Resulting polymer was fluorescently conjugated and used for *in vitro* and *in vivo* characterization of cell binding and uptake.

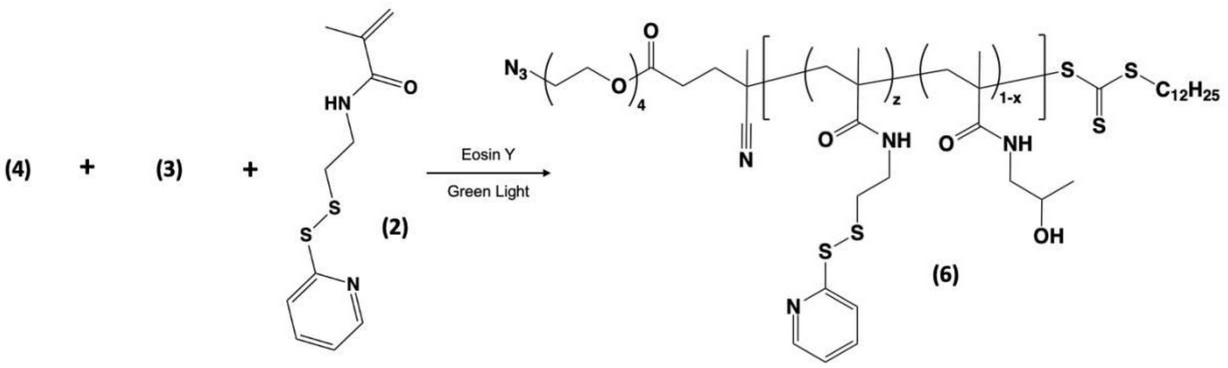

p(PDS) (6). HPMA **(3)** (200 mg, 1.40 mmol), PDS monomer **(2)** (68.6 mg, 0.27 mmol**),** and CTA **(4)** (16.3 mg, 0.027 mmol) were dissolved in 1 mL DMSO in a schlenk tube. Eosin Y (0.374 mg, 0.00054 mmol) was added in 50 μL DMSO. The tube was sealed and degassed via four freeze-pump thaw cycles then placed inside a foil-wrapped bowl with green LED strip lights. The reaction was covered with foil and left stirring for 14 hours. After that time, the polymer was precipitated in cold diethyl ether three times to remove residual monomer. The resulting polymer (150 mg) was dried under reduced pressure and characterized with ^{1}H NMR and GPC using PMMA standards. The p(PDS) used in binding studies had a number averaged molecular weight of 8,197 and a dispersity of 1.387. Using ^{1}H NMR, it was determined that the polymer had an average of 8.0 PDS monomers per chain. The low PDS polymer used in the *in vivo* binding experiment was synthesized with half of the PDS monomer, with HPMA replaced in a weight equivalent. This polymer had a number averaged molecular weight of 7,972 and a dispersity of 1.255, with 3.1 PDS monomers per chain. Resulting polymer was fluorescently conjugated and used for *in vitro* and *in vivo* characterization of cell binding and uptake.

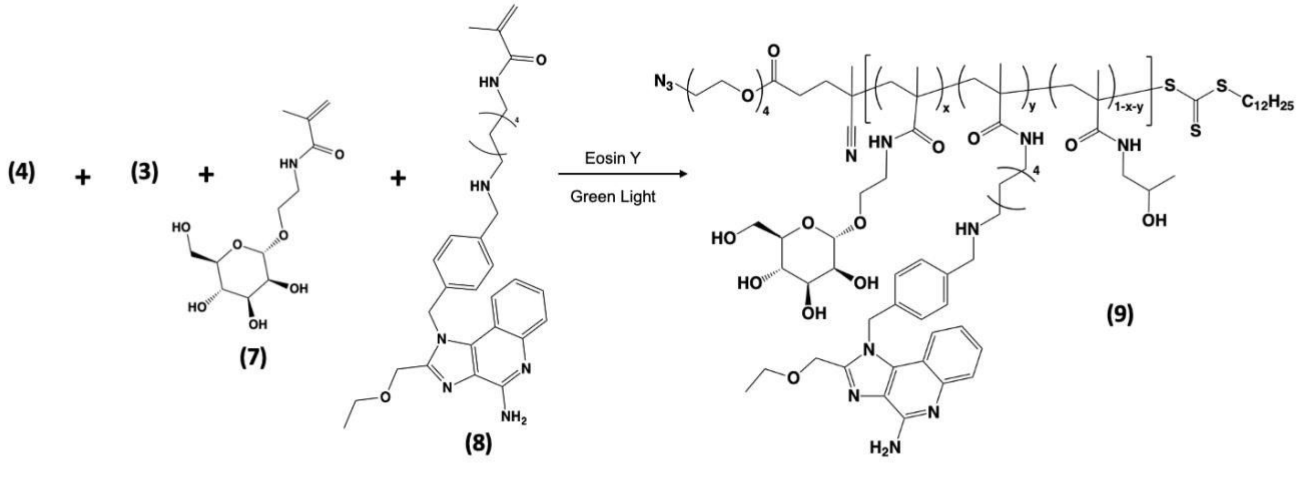

p(Man-TLR7) (9). HPMA **(3)** (68.4 mg, 0.478 mmol), mannose monomer **(7)** (43.8 mg, 0.151 mmol), TLR7 monomer **(8)** (40 mg, 0.70 mmol), and CTA **(4)** (4.2 mg, 0.0069 mmol) were dissolved in 1 mL DMSO in a schlenk tube. Eosin Y (0.09 mg, 0.00013 mmol) was added in 50 μL DMSO. The tube was sealed and degassed via four freeze-pump thaw cycles then placed inside a foil-wrapped bowl with green LED strip lights. The reaction was covered with foil and left stirring for 14 hours. After that time, the polymer was precipitated in cold diethyl ether three times to remove residual monomer. The resulting polymer (70 mg) was dried under reduced pressure and characterized with ^{1}H NMR and GPC using PMMA standards. Representative NMR spectrum is shown below, where unique monomer peaks were identified to quantify their relative abundance. Resulting polymer was used as a non-binding control in *in vitro* and *in vivo* activity and retention experiments.

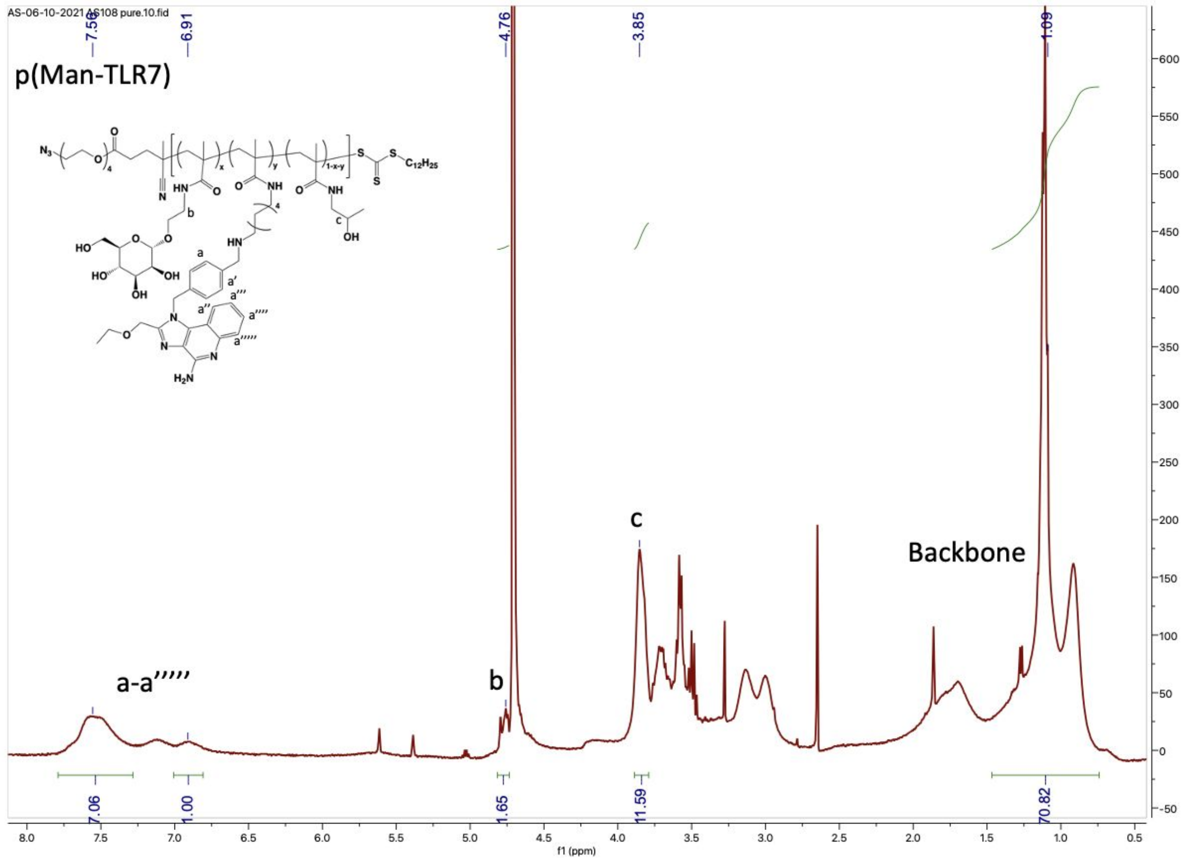

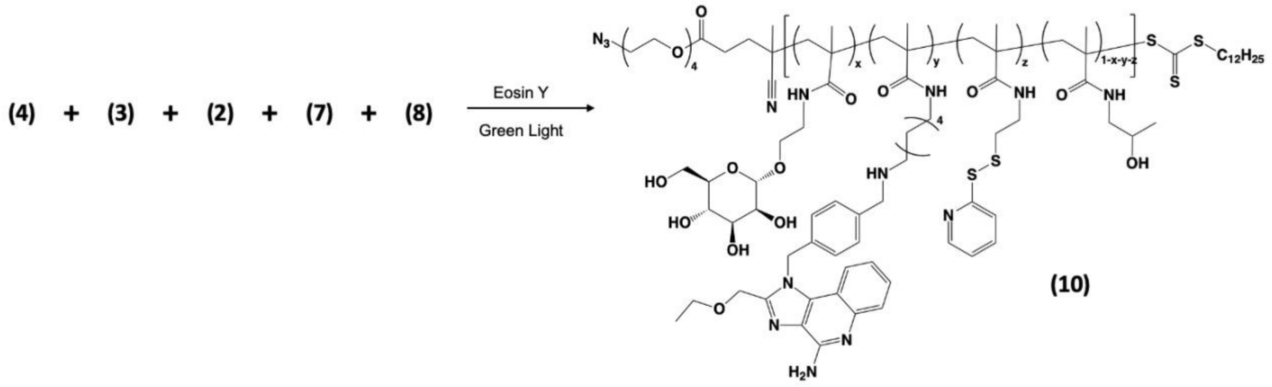

p(Man-TLR7-PDS) (10). HPMA **(3)** (56.5 mg, 0.395 mmol), PDS monomer **(2)** (19.9 mg, 0.079 mmol), mannose monomer **(7)** (49.3 mg, 0.169 mmol), TLR7 monomer **(8)** (45 mg, 0.078 mmol), and CTA **(4)** (4.8 mg, 0.0079 mmol) were dissolved in 1 mL DMSO in a schlenk tube. Eosin Y (0.102 mg, 0.00015 mmol) was added in 50 μL DMSO. The tube was sealed and degassed via four freeze-pump thaw cycles then placed inside a foil-wrapped bowl with green LED strip lights. The reaction was covered with foil and left stirring for 14 hours. After that time, the polymer was precipitated in cold diethyl ether three times to remove residual monomer. The resulting polymer (100 mg) was dried under reduced pressure and characterized with ^1H NMR and GPC using PMMA standards. Representative NMR spectrum is shown below, where unique monomer peaks were identified to quantify their relative abundance. Resulting polymer was used in *in vitro* and *in vivo* activity and retention experiments.

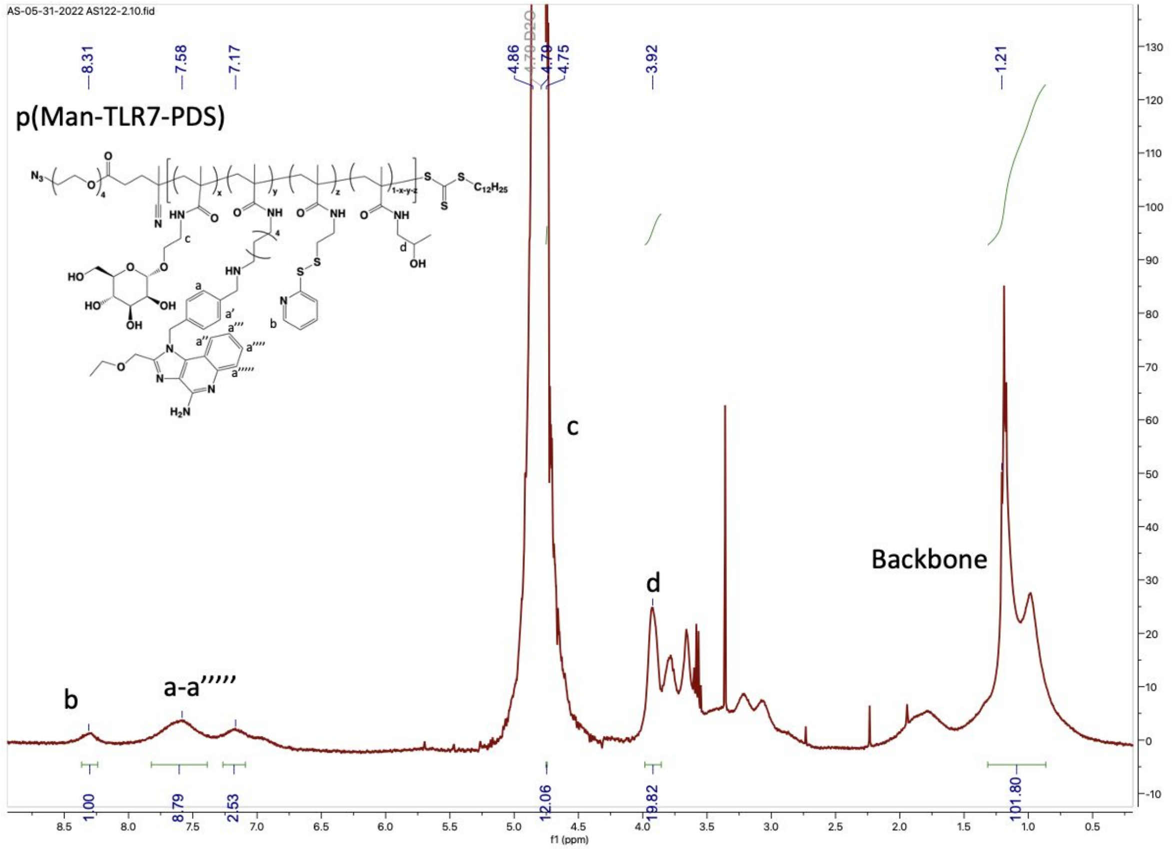

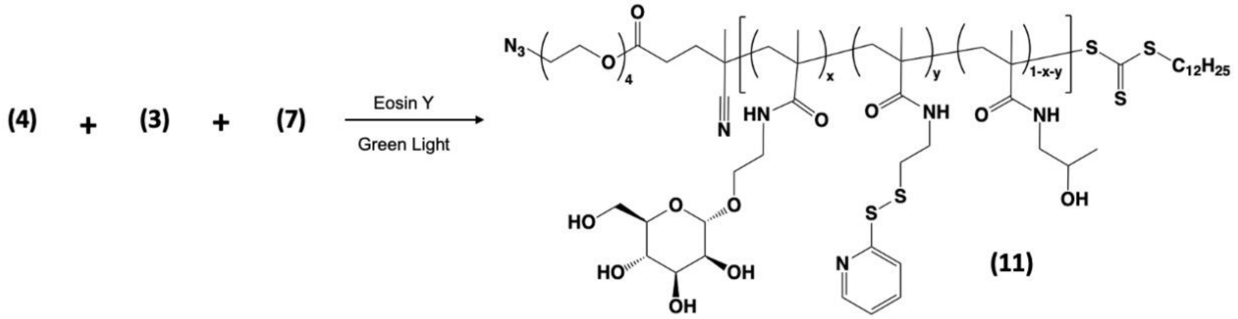

(11)

p(Man-PDS) (11). HPMA **(3)** (100 mg, 0.699 mmol), PDS monomer **(2)** (35.3 mg, 0.139 mmol), mannose monomer **(7)** (87.2 mg, 0.299 mmol) and CTA **(4)** (8.4 mg, 0.014) mmol) were dissolved in 1 mL DMSO in a schlenk tube. Eosin Y (0.18 mg, 0.00028 mmol) was added in 50 µL DMSO. The tube was sealed and degassed via four freeze-pump thaw cycles then placed inside a foil-wrapped bowl with green LED strip lights. The reaction was covered with foil and left stirring for 14 hours. After that time, the polymer was precipitated in cold diethyl ether three

times to remove residual monomer. The resulting polymer (100 mg) was dried under reduced pressure and characterized with ^{1}H NMR and GPC using PMMA standards. Representative NMR spectrum is shown below, where unique monomer peaks were identified to quantify their relative abundance. Resulting polymer was used as a non-adjuvanted control in *in vitro* and *in vivo* activity experiments and was fluorescently conjugated and used for *in vitro* characterization of cell binding and uptake.

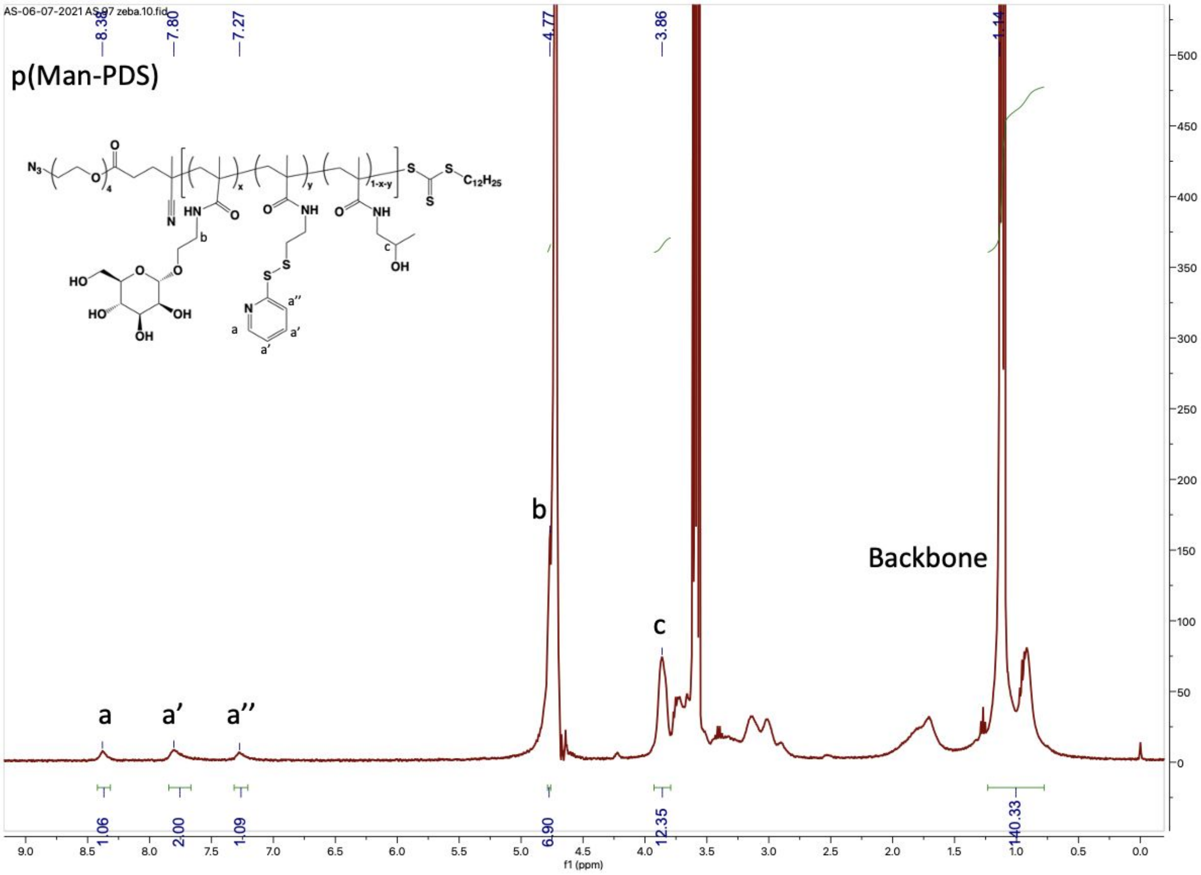

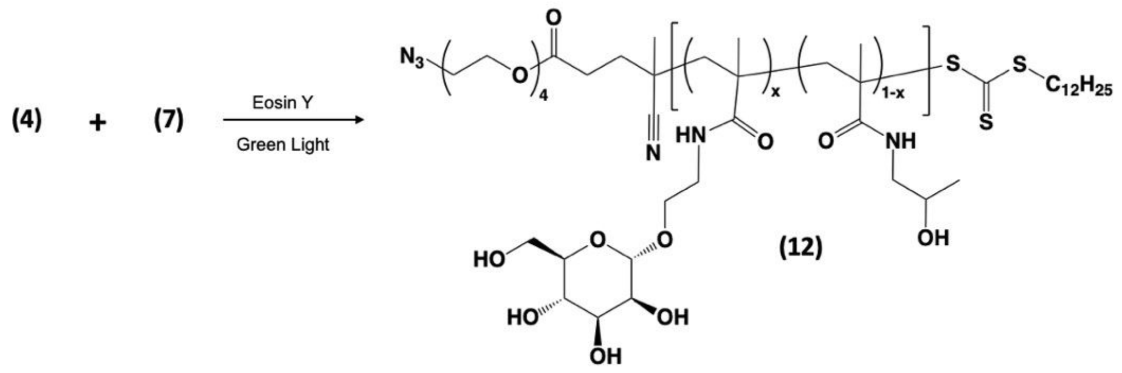

p(Man) (12). HPMA **(3)** (104 mg, 0.727 mmol), mannose monomer **(7)** (59.1 mg, 0.202 mmol) and CTA **(4)** (7.6 mg, 0.013) mmol) were dissolved in 1 mL DMSO in a schlenk tube. Eosin Y (0.16 mg, 0.00025 mmol) was added in 50 µL DMSO. The tube was sealed and degassed via four freeze-pump thaw cycles then placed inside a foil-wrapped bowl with green LED strip lights. The reaction was covered with foil and left stirring for 14 hours. After that time, the polymer was precipitated in cold diethyl ether three times to remove residual monomer. The resulting polymer (100 mg) was dried under reduced pressure and characterized with ^{1}H NMR and GPC using PMMA standards. Resulting polymer was fluorescently conjugated and used for *in vitro* characterization of cell binding and uptake.
